# Salicylic acid regulates biosynthesis of floral fragrance (*E*)-β-farnesene via NPR3-WRKY1 module in chrysanthemum

**DOI:** 10.1186/s43897-025-00174-y

**Published:** 2025-09-05

**Authors:** Zhiling Wang, Yixin Yuan, Rui Dong, Ruihong Zeng, Xin Zhao, Yanjie Xu, Junping Gao, Bo Hong, Zhaoyu Gu

**Affiliations:** Beijing Key Laboratory of Development and Quality Control of Ornamental Crops, Department of Ornamental Horticulture, China Agricultural University, Beijing, 100193 China

**Keywords:** Aroma, Biosynthesis, Chrysanthemum, (*E*)-β-farnesene, Salicylic acid

## Abstract

Floral fragrance is essential for the attraction of pollinators and responses to biotic and abiotic stresses. It also enhances the quality and economic value of plants. Phytohormones, acting as key signaling molecules, are crucial roles in regulating plant growth and development. However, the molecular mechanisms underlying the biosynthesis of fragrance-related volatiles and their crosstalk with other endogenous signals within plants remain largely unknown. Here, we identified a sesquiterpene synthase gene, *CmEβFS*, in chrysanthemum, which encodes a catalytic enzyme responsible for synthesizing the key fragrance-related compound (*E*)-β-farnesene. We demonstrated that *CmEβFS* is negatively regulated by CmWRKY1, thereby inhibiting (*E*)-β-farnesene biosynthesis. Furthermore, CmWRKY1 interacts with the salicylic acid (SA) receptor CmNPR3 to regulate SA-mediated *CmEβFS* transcription. Our findings reveal that SA regulates (*E*)-β-farnesene biosynthesis by controlling *CmEβFS* expression via the CmWRKY1-CmNPR3 module during floral development. These findings enhanced our understanding of the mechanisms underlying SA-mediated regulation of volatile organic compounds (VOCs) biosynthesis and provided insights into improving floral fragrance.

**Supplementary Information:**

The online version contains supplementary material available at 10.1186/s43897-025-00174-y.

## Core

The study demonstrates the *CmEβFS* in chrysanthemum, which encodes an enzyme for (*E*)-β-farnesene synthesis, a key floral fragrance compound. *CmEβFS* is negatively regulated by CmWRKY1-CmNPR3 module, in response to salicylic acid (SA). This finding provides valuable insights into the mechanisms of phytohormone-mediated VOC biosynthesis and offers potential strategies for improving floral fragrance.

## Gene & accession numbers

The sequence data accession numbers used in this study can be found in the National Center for Biotechnology Information databases as follows: *CmEβFS* (PQ588680), *CmWRKY1* (PQ588682) and *CmNPR3* (PQ588684).

## Introduction

In flowering plants, aroma serves many vital biological functions, such as attracting pollinators (Byers 2021), facilitating plant-to-plant communication (Grandi et al. 2024), responding to environmental changes (Keefover-Ring et al. 2022), and defending against pathogens and herbivores (Sobhy and Bruce 2021; Brosset and Blande 2022). Floral fragrance is determined by the composition and concentration of volatile organic compounds (VOCs) (Dudareva et al. 2013; Peng et al. 2025). VOCs are classified into three categories based on their biosynthetic origins: terpenoids, phenylpropanoid/benzenoid compounds, and fatty acid derivatives (Koeduka 2024). Terpenoids (terpenes or isoprenoids) form the largest and most diverse class of secondary metabolites in plants (Jiang et al. 2023). In chrysanthemums (*Chrysanthemum* × *morifolium*) and their wild relatives, the primary VOCs consist of monoterpenoids, including amphene, sabinene, and 1,8-cineole, and sesquiterpenoids, such as (*E*)-β-farnesene, germacrene D, β-ylangene, and β-copaene (Zhang et al. 2021; 2024). In rose (*Rosa rugosa*), the characteristic floral scent is primarily attributed to key terpene compounds such as geraniol, nerol, and citronellol, which also serve as the main constituents of rose essential oil (Shang et al. 2024).

Terpenoid biosynthesis occurs through two pathways: the mevalonate pathway, which produces sesquiterpenes and triterpenes in the cytosol, and the methylerythritol phosphate pathway, which generates monoterpenes, diterpenes, and tetraterpenes in plastids (Daletos et al. 2020). The key enzymes in the final step of terpenoid biosynthesis are terpene synthases (TPSs), which convert the substrates geranyl diphosphate (GPP) and farnesyl diphosphate (FPP) into monoterpenes and sesquiterpenes in plastids and the cytosol, respectively (Bao et al. 2020; 2023). Currently, the *TPS* gene family comprises over 100 members identified from various plant species (Bergman and Dudareva 2024; Li et al. 2024a). The activity of TPSs contributes to the diversity of VOCs in various tissues and developmental stages of plants (Gao et al. 2018; Bao et al. 2023).

Transcription factors (TFs) play a crucial role in regulating the expression of *TPSs* and modulating terpenoid biosynthesis. In *Freesia hybrida*, the MYB-bHLH complex positively regulates *FhTPS1* expression, thereby promoting linalool biosynthesis (Yang et al. 2020). In cotton (*Gossypium arboreum*), GaWRKY1 positively regulates sesquiterpene biosynthesis by controlling the expression of the (+)-*δ*-cadinene synthase gene *CAD1-A* (Xu et al. 2004). In chrysanthemum, CmWRKY41 regulates sesquiterpenes biosynthesis by activating the expression of *3-hydroxy-3-methylglutaryl-CoA reductase 2* (*CmHMGR2*) and *farnesyl pyrophosphate synthase 2* (*CmFPPS2*) (Hu et al. 2023). These findings revealed that TFs are essential for regulating the synthesis of terpenoids. However, it remains largely unclear which upstream components might influence the transcriptional activation of TFs. Furthermore, it is also uncertain whether additional endogenous signals co-regulate the accumulation of terpenoids in plants.

Plant hormones play critical roles in terpenoid biosynthesis. In *Artemisia annua*, jasmonic acid (JA) and abscisic acid (ABA) regulate artemisinin biosynthesis through AabHLH113 (Yuan et al. 2023). Methyl jasmonate (MeJA) treatment increased artemisinin content in *Artemisia annua* suspension cell cultures (Baldi and Dixit 2008). In sweet orange (*Citrus sinensis*), ethylene enhanced the sesquiterpene (+)-valencene biosynthesis (Shen et al. 2016). Salicylic acid (SA) is a crucial plant hormone involved in plant metabolism, homeostasis, and growth (Zhang and Li 2019; Ding and Ding 2020). Nonexpresser of pathogenesis-related (NPR) proteins function as SA receptor, facilitating the reprogramming of large-scale gene expression triggered by SA. Among them, NPR1 and NPR2 function as positive regulators of downstream genes in response to SA, whereas NPR3 and NPR4 act as negative regulators (Ding and Ding 2020; Jia et al. 2023). While research on SA has primarily focused on its roles in enhancing disease resistance and promoting plant growth and development (Vicente and Plasencia 2011; Van Butselaar and Van den 2020; Zavaliev and Dong 2024). However, the mechanism of SA in regulating the formation of floral aroma in plants remains largely unexplored.

Chrysanthemums are globally important floral plants with distinct scents and valuable uses as cut flowers, garden plants, medicinal herbs, and edible plants, including teas and fresh greens (Zou et al. 2021; Zhong et al. 2022). Chrysanthemums belong to the Asteraceae family and are characterized by high ploidy and high heterozygosity (Song et al. 2023a), contributing to their extensive variation in scent types. In our previous study, we categorized chrysanthemum scent types into six categories and identified (*E*)-β-farnesene is a conserved volatile component in the fruit-scented genetic group (Wang et al. 2023).

(*E*)-β-farnesene, a sesquiterpene, is an important aromatic compound and a key component of essential oils (An et al. 2025; Tai et al. 2025), also serving as an aphid alarm pheromone (Li et al. 2019). Current research on (*E*)-β-farnesene primarily focuses on two aspects: its ecological role in aphid defense, either through direct release or by attracting natural enemies, such as in in pyrethrum (*Tanacetum cinerariifolium*) (Li et al. 2019), and its quantification as an important aroma component in plants such as chrysanthemum (Zhang et al. 2021) and chamomile (*Matricaria recutita*) (Rawat et al. 2022). The biosynthesis of (*E*)-β-farnesene is catalyzed of (*E*)-β-farnesene synthase gene (*EβFS*). Several *EβFS* have been isolated from various plant species, including German chamomile (*Matricaria chamomilla* L.) (Tai et al. 2025) and silk tree (*Albizia julibrissin*) (Liu et al. 2021). Although *EβFS* is recognized as the synthase gene responsible for (*E*)-β-farnesene synthesis, its transcriptional regulation in plants remains largely unexplored.

Here, we reported that (*E*)-β-farnesene acts as a significant floral fragrance substance in chrysanthemum under SA-mediated modulation. CmEβFS is identified as the key catalytic enzyme responsible for (*E*)-β-farnesene production. *CmEβFS* expression is negatively regulated by CmWRKY1. Furthermore, the CmNPR3-CmWRKY1 module functions as a negative regulator of *CmEβFS* response to SA in chrysanthemum. Our findings provided valuable insights into the molecular mechanisms underlying the integration of plant hormone signal transduction and the biosynthesis of VOCs.

## Results

### (*E*)-β-farnesene is the most abundant volatile compound in chrysanthemum

**Fig. 1:**
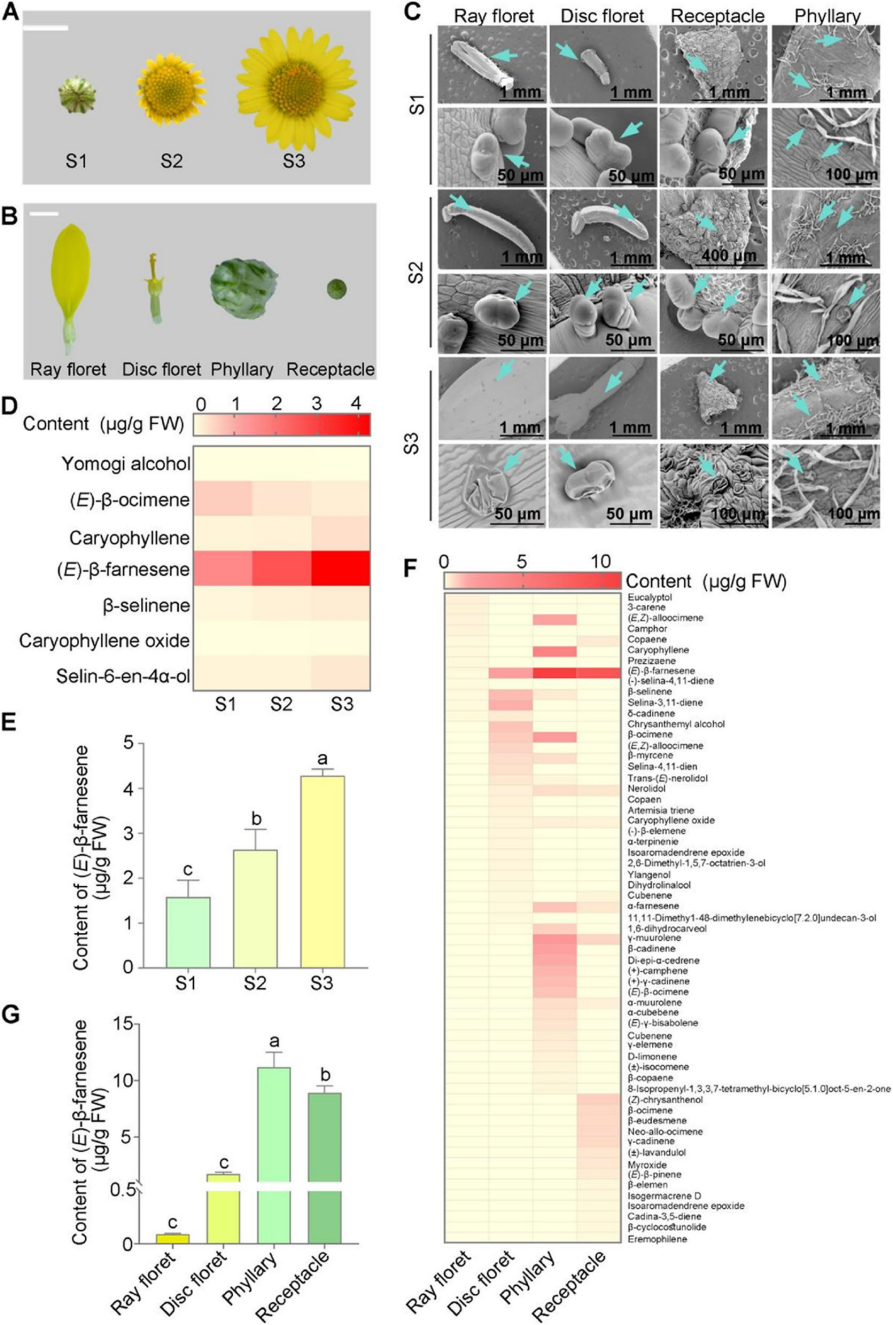
Spatiotemporal analysis of (*E*)-β-farnesene production during floral development in chrysanthemum. **A** Different floral development stages of chrysanthemum. Stage 1, the flower bud stage; Stage 2, the initial flower stage with ray florets that have not yet fully opened; Stage 3, the blooming stage with ray florets fully opened. Scale bar, 1 cm. **B** Four floral organs were separated from stage 3 flowers: ray floret, disc floret, phyllary, and receptacle. Scale bar, 2 mm. **C** Glandular trichomes development in different organs of chrysanthemum at stage 1–3. The top row of each diagram in every stage shows the status of glandular trichomes in different organs, while the second row exhibits the status of individual glandular trichomes within each organ. The arrows indicate the positions of the glands. **D** Dynamic changes of volatile terpenoids content during floral development. The content of seven terpenoids during floral development. The content of all the terpenoids analysis in the three floral development stages were shown in Fig. S2. The color scale was used to indicate the content of the VOCs (μg/g FW). **E** (*E*)-β-farnesene content during floral development stages. **F** Volatile terpenoids content in different floral organs at stage 3. The color scale was used to indicate the content of the VOCs (μg/g FW). **G** (*E*)-β-farnesene content in different floral organs. Data in **E**) and **G**) represent the mean ± SD of three biological replicates (*n* = 3). Statistical significance was assessed using one-way ANOVA with the Tukey comparisons test (*P* < 0.05), with different letters representing statistically significant differences. For **D**), **E**), each flower is one biological replicate; mean ± SD is shown from three biological replicates (*n* = 3). For **F**) and** G**), the ray florets, disc florets, phyllaries, receptacles of 20 flowers were pooled together as one biological replicate. S1, Stage 1; S2, Stage 2; S3, Stage 3

We previously showed that terpenoids are the primary volatile compounds contributing to the fragrance of fruit-scented chrysanthemums (Wang et al. 2023). To further investigate the synthesis mechanism of volatiles, we categorized the floral developmental process into three stages: the flower bud stage (stage 1), the initial flower stage (stage 2), and the blooming stage (stage 3) (Fig. 1A). Meanwhile, we separated flowers into four organs: ray floret, disc floret, phyllary, and receptacle (Fig. 1B). Through scanning electron microscopy (SEM) revealed that glandular trichomes were present exclusively in the ray florets, disc florets and receptacles, while both glandular trichomes and T-shaped trichomes were observed in the phyllaries (Fig. 1C). Additionally, trichome density was significantly higher in phyllaries and receptacles than in ray and disc florets across all stages of floral development (Fig. 1C). Specifically, the glandular trichomes in stage 1 receptacles were smooth and spherical, while those in stage 3 receptacles displayed varying degrees of cracking. Furthermore, we quantified the number of ruptured and unruptured glandular trichomes in different floral organs across all developmental stages. The results showed that stages 1 and 2 exhibited a higher proportion of unruptured glandular trichomes in all floral organs, whereas stage 3 displayed a significant increase in ruptured trichomes (Fig. S1). These findings indicated that the maturation and rupturing of glandular trichome during floral development contributed to an increase in the secretion of VOCs.

Gas chromatography-mass spectrometry (GC–MS) analysis revealed the presence of 24 terpenoids in chrysanthemum flowers (Fig. S2). Notably, seven terpenoids were consistently identified in flowers at all three developmental stages, including yomogi alcohol, (*E*)-β-ocimene, caryophyllene, (*E*)-β-farnesene, β-selinene, caryophyllene oxide, and selin-6-en-4α-ol (Fig. 1D). (*E*)-β-farnesene exhibited significantly higher abundance compared to other terpenoids. Its abundance increased gradually and peaked at stage 3 (Fig. 1D, E). Furthermore, GC–MS analysis also showed that (*E*)-β-farnesene was the most abundant terpenoid in all floral organs at all development stages, particularly in the phyllaries and receptacles (Fig. 1F, G; Fig. S3A, B). Additionally, (*E*)-β-farnesene was detected in glandular trichome extracts (Fig. S4). Therefore, we focused on (*E*)-β-farnesene as a key VOC involved in the formation of chrysanthemum fragrance.

### CmEβFS catalyzes the formation of (*E*)-β-farnesene

**Fig. 2:**
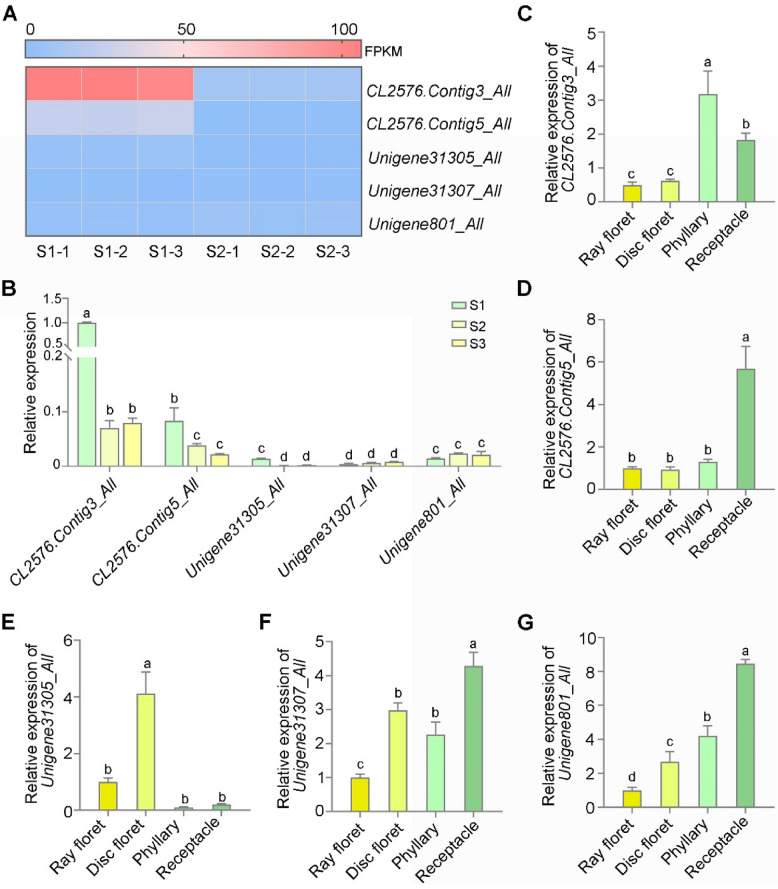
Spatiotemporal expression patterns of (*E*)-β-farnesene synthase genes in chrysanthemum. **A** Expression patterns of five terpene synthase (*TPS*) genes, annotated as (*E*)-β-farnesene synthase genes, were analyzed during different floral development stages by transcriptome analysis. The color scale represents the FPKM values. **B** Expression levels of five candidate genes during the floral development process in chrysanthemum, as determined by RT-qPCR. The five candidate genes were selected by transcriptome data. **C**-**G** Expression levels of five candidate genes in different floral organs during stage 3, as determined by RT-qPCR. *CmUBI* was used as an internal reference. For transcriptome sequence, ten flowers from each developmental stage were pooled together as one biological replicate. Three biological replicates were analyzed in this experiment. For RT-qPCR analysis, five flowers were pooled together as one biological replicate. Values are the means ± SD of three biological replicates (*n* = 3). Statistical significance was determined by one-way ANOVA (**C**-**G**) or two-way ANOVA (B) with the Tukey comparisons test (*P* < 0.05)

(*E*)-β-farnesene levels significantly varied between stages 1 and 2 (Fig. 1E). We selected chrysanthemum flowers at these two stages for transcriptomic sequencing to identify potential synthase-encoding genes responsible for (*E*)-β-farnesene production. Transcriptome data was analyzed (Table S1−3), and 56 *TPSs* were identified. Among them, five were annotated as (*E*)-β-farnesene synthase genes (Fig. 2A, Fig. S5).

The expression levels of the five (*E*)-β-farnesene synthase genes were further verified using reverse transcription-quantitative polymerase chain reaction (RT-qPCR). Consistent with the transcriptome data, *CL2576.Contig3_All* exhibited consistently high expression throughout floral development, with levels significantly higher than those of the other four (*E*)-β-farnesene synthase genes (Fig. 2B). Meanwhile, the expression profiles of the five candidate genes were examined in different floral organs in all floral development stages using RT-qPCR. All five genes were expressed in the four floral organs. However, their expression patterns varied: *CL2576.Contig3_All*, *CL2576.Contig5_All*, *Unigene31305_All*, *Unigene31307_All, *and *Unigene801_All* were predominantly expressed in phyllaries, receptacles, disc florets, receptacles, and receptacles, respectively (Fig. 2C-G; Fig. S6A-J). Notably, the expression patterns of *CL2576.Contig3_All* were consistent with (*E*)-β-farnesene levels in different floral organs but inconsistent with (*E*)-β-farnesene content during floral development. Integrating these findings with glandular trichomes observations, we speculate that the biosynthesis of (*E*)-β-farnesene predominantly occurs during the flower bud stage. As floral development progresses and mature glands continue to rupture, aromatic substances, including (*E*)-β-farnesene, are secreted. This pattern is consistent with previous findings in marijuana (Hammond and Mahlberg 1977; Jorge and Jules 1995).

**Fig. 3:**
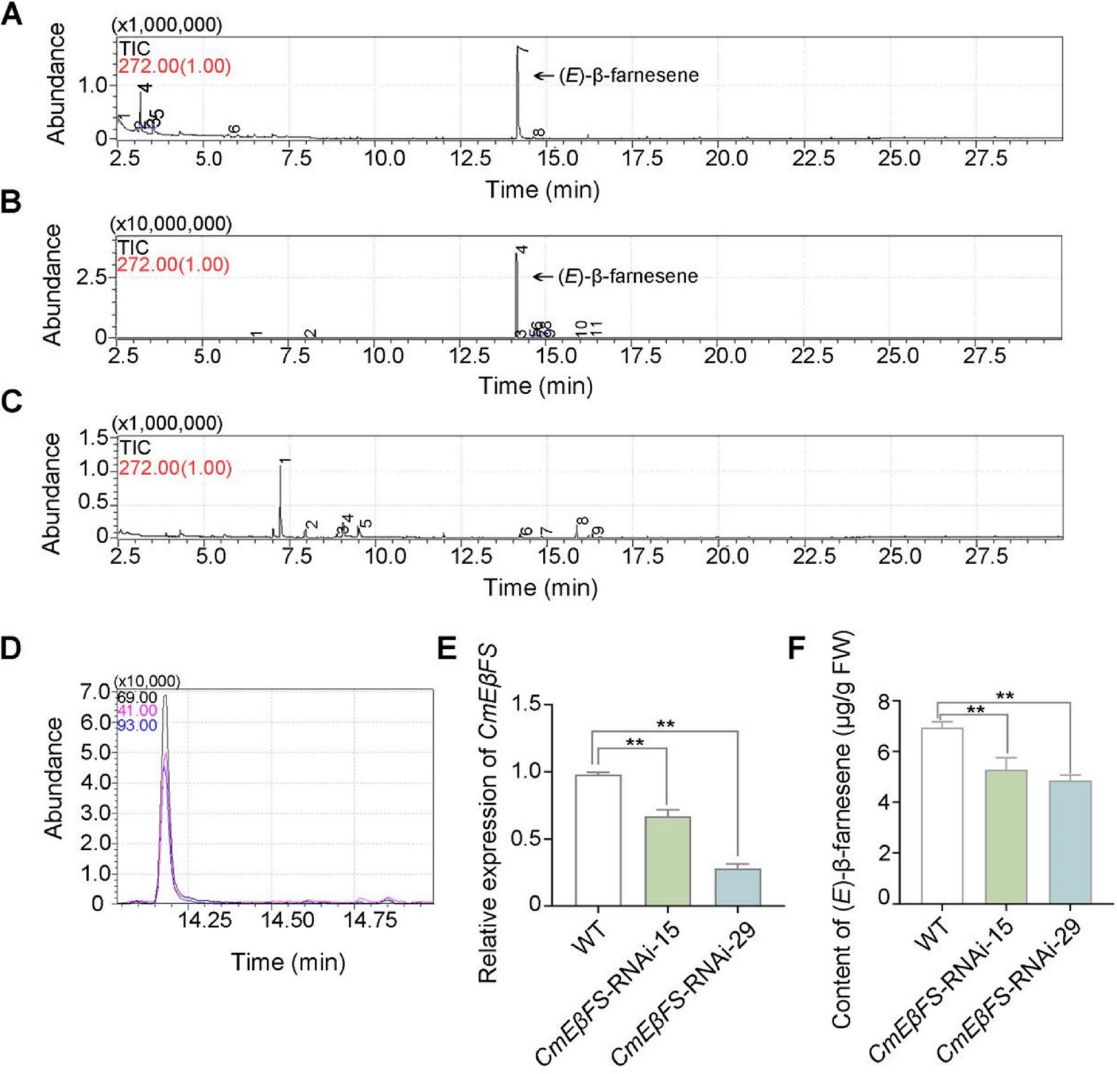
CmEβFS is the catalytic enzyme of (*E*)-β-farnesene **A** Product peaks showing the standard of (*E*)-β-farnesene (CATO Research Chemicals Inc., Guangzhou). **B**-**C** The results of enzyme activity assay in vitro for CmEβFS using FPP (B) or GPP (C) as substrates. FPP, farnesyl diphosphate. GPP, geranyl diphosphate. Recombinant His-CmEβFS proteins purified from *E. coli* were mixed with FPP or GPP. The enzymatic reaction products were analyzed by GC–MS. **D** GC–MS analysis of (*E*)-β-farnesene production catalyzed from FPP as substrates by CmEβFS protein extracted from chrysanthemum flowers using anti-CmEβFS antibodies. The peaks of different colors represent the overlap between the (*E*)-β-farnesene production and the standard samples. **E** RT-qPCR analysis of *CmEβFS* expression in flowers of the WT and *CmEβFS*-RNAi plants. *CmUBI* was quantified and served as the internal control. **F** GC–MS analysis of (*E*)-β-farnesene content in flowers of the WT and *CmEβFS*-RNAi plants. WT, wild type. For RT-qPCR analysis in **(E)**, five flowers at stage 3 were pooled together as one biological replicate. For GC–MS analysis in **(F)**, one flower at stage 3 was collected as one biological replicate. Values are the means ± SD of three biological replicates (*n* = 3). Statistical significance was determined by Student’s *t*-test (**P < 0.01)

As the expression levels of *CL2576.Contig3_All* correlated with (*E*)-β-farnesene content in all floral organs during floral development, we hypothesized that *CL2576.Contig3_All* might be the key gene responsible for (*E*)-β-farnesene production in chrysanthemum. Multiple sequence alignment analysis revealed that CL2576.Contig3_All contains a conserved DDxxD domain, a hallmark of TPS proteins (Fig. S7A). Phylogenetic analysis showed that CL2576.Contig3_All was closely related to EβFS from sweet wormwood (AaEβFS) (Fig. S7B). Thus, we named CL2576.Contig3_All as CmEβFS. Furthermore, subcellular localization analysis showed that CmEβFS-GFP fusion protein did not co-localize with chloroplasts in transiently transformed *Nicotiana benthamiana* leaf cells, indicating that it mainly functions in the cytosol (Fig. S7C). To further assess the function of CmEβFS, its recombinant protein was prepared (Fig. S8). We performed an in vitro enzymatic assay, with (*E*)-β-farnesene standard as the positive control (Fig. 3A). CmEβFS was found to catalyze the conversion of FPP to (*E*)-β-farnesene (Fig. 3B); however, when GPP was used as the substrate, (*E*)-β-farnesene was not synthesized (Fig. 3C). Additionally, CmEβFS was isolated from stage 1 using an anti-CmEβFS antibody and incubated with FPP as the substrate. The results confirmed that CmEβFS converted FPP into (*E*)-β-farnesene (Fig. 3D). Taken together, these results indicated that CmEβFS functions as the key synthetase during (*E*)-β-farnesene production.

To further elucidate the biological function of *CmEβFS* in chrysanthemum, we specifically knocked down *CmEβFS* by generating corresponding *CmEβFS*-RNA interference (RNAi) lines. We assessed the relative expression levels of *CmEβFS* using RT-qPCR and found that *CmEβFS* expression was significantly reduced in *CmEβFS*-RNAi transgenic plants compared to untransformed wild-type (WT) (Fig. 3E). Additionally, we examined the expression levels of the four other (*E*)-β-farnesene synthase genes identified in the transcriptome data and found no significant differences between WT and transgenic plants (Fig. S9). Furthermore, GC–MS analysis revealed a significant decrease in (*E*)-β-farnesene content in the transgenic plants compared to WT (Fig. 3F). These findings indicated that CmEβFS functions as a catalytic enzyme responsible for (*E*)-β-farnesene biosynthesis in chrysanthemum.

### CmWRKY1 inhibits (*E*)-β-farnesene biosynthesis by negatively regulating *CmEβFS* expression

To identify the potential upstream regulators of *CmEβFS*, we performed a yeast one-hybrid (Y1H) screen with different *CmEβFS* promoter regions and a yeast cDNA library prepared from chrysanthemum flowers. Seven transcription factors were identified in this screening process (Table S4). Among these, CL6014.Contig2_All exhibited strong binding to the *CmEβFS* promoter in the Y1H assay (Fig. S10A). Expression analysis revealed that *CL6014.Contig2_All* was significantly upregulated during floral development, with a rapid increase compared to stage 1 (Fig. S10B). Phylogenetic analysis showed that CL6014.Contig2_All is closely related to Arabidopsis WRKY1 (Fig. S11A). Therefore, CL6014.Contig2_All could be named CmWRKY1. To determine subcellular localizations, we transiently co-expressed the nuclear marker H2B-mCherry with CmWRKY1-GFP in *N. benthamiana* leaves. CmWRKY1-GFP co-localized with H2B-mCherry (Fig. S11B), indicating that CmWRKY1 is primarily localized in the nucleus.

**Fig. 4:**
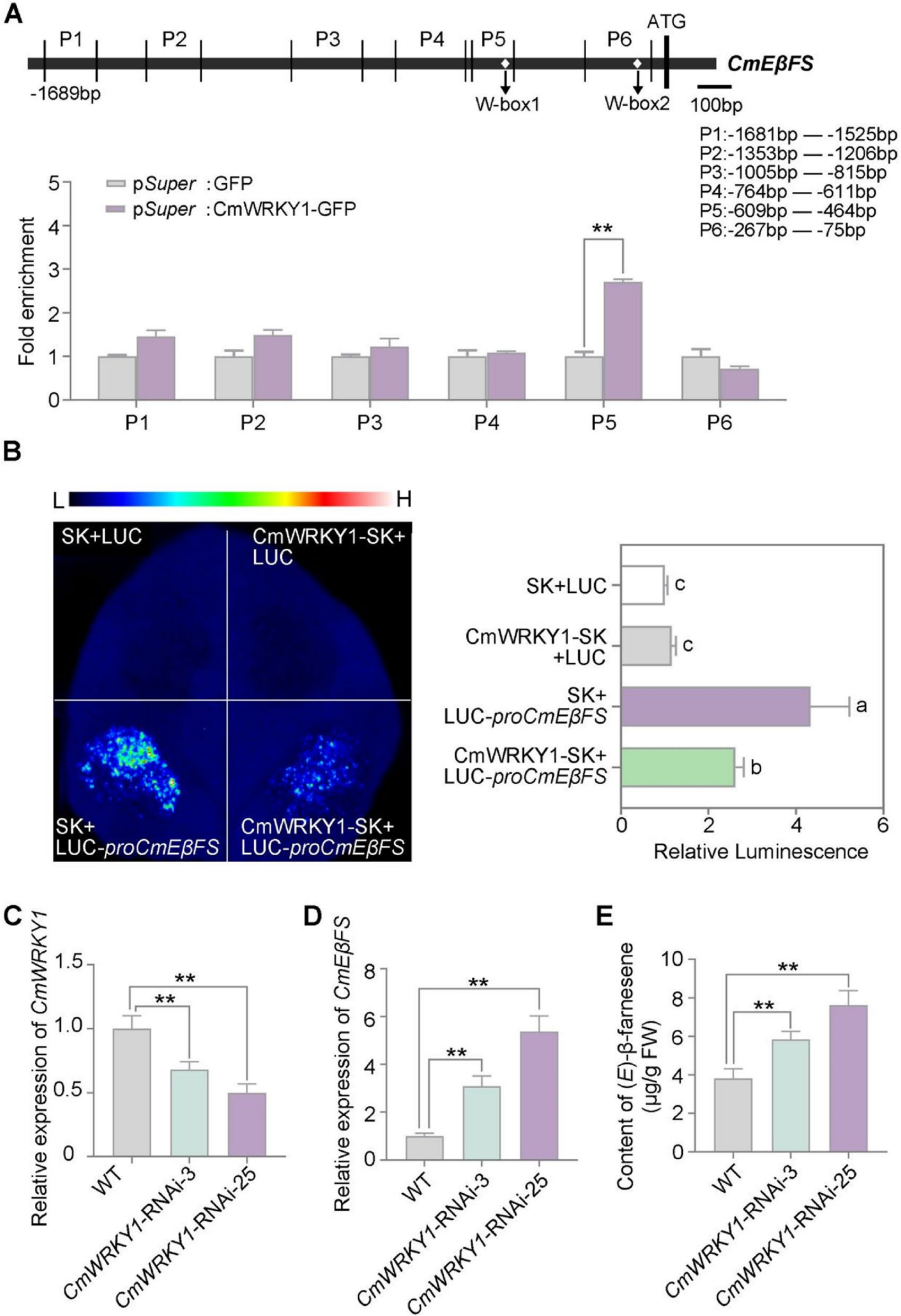
CmWRKY1 negatively regulates *CmEβFS* expression in chrysanthemum, inhibiting (*E*)-β-farnesene biosynthesis. **A** ChIP-qPCR analysis of the indicated fragments (P1-P6) of *CmEβFS* promoter. Chromatin from *pSuper:* CmWRKY1-GFP chrysanthemum plants was immunoprecipitated with an anti-GFP antibody. The *pSuper:* GFP chrysanthemum plants served as a negative control. The amount of the indicated DNA fragment was determined by qPCR and normalized to the *pSuper:* GFP control. **B** Dual-luciferase (LUC) analysis showed that CmWRKY1 inhibited transcription from the *CmEβFS* promoter in *N. benthamiana* leaves (left). Normalized LUC activities are shown as LUC/REN ratios (right). Data represent the mean ± SD of three biological replicates (*n* = 3). Statistical significance was assessed using one-way ANOVA analysis of variance (*P* < 0.05). **C**-**D** RT-qPCR analysis of *CmWRKY1* and *CmEβFS* expression in flowers of the WT and *CmWRKY1*-RNAi plants. *CmUBI* was quantified and served as the internal control. **E** GC–MS analysis of (*E*)-β-farnesene content in flowers of the WT and *CmWRKY1*-RNAi plants. WT, wild type. For RT-qPCR analysis, five flowers at stage 3 were pooled together as one biological replicate. For GC–MS analysis in **(E)**, one flower at stage 3 was collected as one biological replicate. Values are the means ± SD of three biological replicates. For (**C**)-(**E**), statistical significance was determined by Student’s *t*-test (**P < 0.01)

To examine the regulatory relationship between CmWRKY1 and *CmEβFS*, we performed a chromatin immunoprecipitation-quantitative polymerase chain reaction (ChIP-qPCR) assay using an anti-GFP antibody and chrysanthemums overexpressing CmWRKY1-GFP. The results revealed that CmWRKY1 bound to the W-box motif of the *CmEβFS* promoter (Fig. 4A). Furthermore, the results of dual-luciferase (LUC) assays indicated that CmWRKY1 functions as a transcriptional repressor, negatively regulating the promoter activity of *CmEβFS* (Fig. 4B). To better elucidate the function of CmWRKY1 in chrysanthemum, we specifically knocked down *CmWRKY1* by generating corresponding *CmWRKY1*-RNAi lines. RT-qPCR analysis confirmed that *CmWRKY1* expression was significantly reduced in the *CmWRKY1*-RNAi plants compared to WT (Fig. 4C). To confirm the specificity of *CmWRKY1* silencing, four additional homologous genes, *CL14734.Contig1_All*, *CL4741.Contig1_All*, *CL4741.Contig4_All*, and *CL1528.Contig1_All*, which were clustered with *CmWRKY1* based on phylogenetic analysis (Fig. S11C), were examined using RT-qPCR. The expression levels of the four genes were not significantly different between the *CmWRKY1* -RNAi plants and WT (Fig. S11D–G). Compared to WT, the transcript levels of *CmEβFS* were significantly higher in the *CmWRKY1*-RNAi plants (Fig. 4D) and (*E*)-β-farnesene content was significantly higher in the *CmWRKY1*-RNAi plants than in WT (Fig. 4E). These results indicated that CmWRKY1 inhibits (*E*)-β-farnesene biosynthesis by negatively regulating *CmEβFS* expression.

### CmWRKY1 interacts with CmNPR3 to negatively regulate *CmEβFS* transcription

**Fig. 5:**
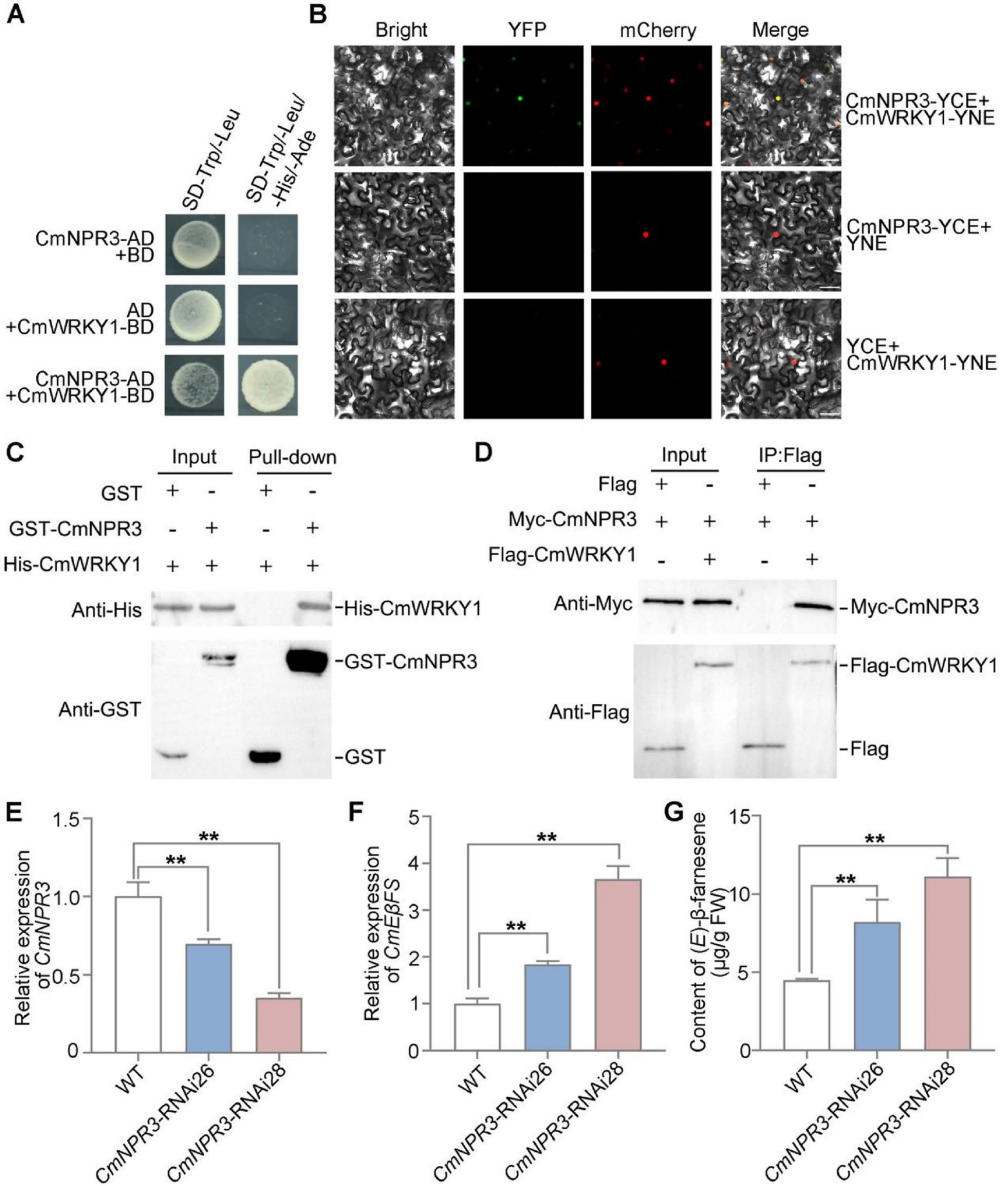
CmNPR3 interacts with CmWRKY1 to repress the biosynthesis of (*E*)-β-farnesene. **A** Interaction between CmWRKY1 and CmNPR3 by Y2H assays. The pGADT7 (AD) and pGBKT7 (BD) empty vectors served as negative controls. Yeast cells were selected on synthetic dropout medium (SD/–Trp–Leu or SD/–Trp/–Leu/–His/–Ade). **B** Interaction between CmWRKY1 and CmNPR3 by BiFC in *N. benthamiana* leaves. CmWRKY1-YNE and CmNPR3-YCE were co-transformed into *N. benthamiana* leaves. The combinations of CmNPR3-YCE and YNE, YCE and CmWRKY1-YNE were used as negative controls. All the combinations were co-expressed with the nucleus marker H2B-mCherry. YFP signals were observed under 488 nm excitation, and mCherry signals were observed under 561 nm excitation. **C** Pull-down assay showing that CmNPR3 interacts with CmWRKY1. Recombinant GST or GST-CmNPR3 bounded to Glutathione Sepharose beads was incubated with recombinant His-CmWRKY1 protein and immunoblotted with anti-His and anti-GST antibody. **D** Co-immunoprecipitation assay showing the interactions between CmNPR3 and CmWRKY1. Myc-CmNPR3 was co-infiltrated with Flag-CmWRKY1 in *N. benthamiana* leaves. Three days after infiltration, total proteins were extracted, and the soluble fraction was immunoprecipitated using an anti-Flag antibody. The precipitated complexes were then analyzed by immunoblotting with both anti-Flag and anti-Myc antibodies. **E**–**F** RT-qPCR analysis of *CmNPR3* and *CmEβFS* expression in flowers of the WT and *CmNPR3*-RNAi plants. *CmUBI* was quantified and served as the internal control. **G** GC–MS analysis of (*E*)-β-farnesene content in flowers of the WT and *CmNPR3*-RNAi plants. WT, wild type. For RT-qPCR analysis, five flowers at stage 3 were pooled together as one biological replicate. For RT-qPCR analysis, five flowers at stage 3 were pooled together as one biological replicate. For GC–MS analysis in **(G)**, one flower at stage 3 was collected as one biological replicate. Values are the means ± SD of three biological replicates. For (**E**)-(**G**), statistical significance was determined by Student’s *t*-test (**P < 0.01)

To elucidate the regulatory mechanisms underlying the regulation of (*E*)-β-farnesene biosynthesis by CmWRKY1 during floral development, we performed a pull-down assay followed by mass spectrometry. CmWRKY1-His protein was incubated with crude proteins extracted from chrysanthemum flowers to identify its potential interactors. Mass spectrometry revealed that 38 proteins that interacted with CmWRKY1 (Table S5). Among them, we focused on the protein annotated as salicylic acid receptor. Unigene43188_All, which interacts with CmWRKY1, has been annotated as an SA receptor-associated protein NPR3. Phylogenetic analysis showed that Unigene43188_All was closely related to NPR3 from sweet wormwood (AaNPR3) (Fig. S12A). Hence, Unigene43188_All was named as CmNPR3. Subcellular localization assays showed that the fusion protein CmNPR3-GFP co-localized with H2B-mCherry in the nucleus in *N. benthamiana* leaves (Fig. S12B). To validate the interaction between CmWRKY1 and CmNPR3, we performed a yeast two-hybrid (Y2H) assay. The results also indicated that CmWRKY1 interacted with CmNPR3 (Fig. 5A). Combining the results of Y2H, we concluded that CmWRKY1 interacted with CmNPR3 both in in vitro and in vivo. These results were further supported by bimolecular fluorescence complementation (BiFC) assays, where fluorescence from yellow fluorescent protein (YFP) was detected in the CmWRKY1-CmNPR3 combination (Fig. 5B). Additionally, pull-down and Co-IP assays further confirmed the interaction between CmWRKY1 and CmNPR3 (Fig. 5C, D). These findings indicated that CmWRKY1 physically interacts with CmNPR3.

To assess the biological function of CmNPR3 in chrysanthemum, we silenced *CmNPR3* by generating *CmNPR3*-RNAi lines in chrysanthemum. RT-qPCR analysis confirmed that *CmNPR3* expression was significantly reduced in the *CmNPR3*-RNAi plants than in WT (Fig. 5E). To further validate *CmNPR3* silencing, additional homologous gene, *CL10577.Contig2_All*, which was clustered with *CmNPR3* based on phylogenetic analysis (Fig. S12A), was examined using RT-qPCR. The expression levels of *CL10577.Contig2_All*, was not significantly different between the *CmNPR3*-RNAi plants and WT (Fig. S12C). Compared to WT, *CmEβFS* transcript levels were significantly higher in the *CmNPR3*-RNAi plants (Fig. 5F), and GC–MS analysis revealed a significant increase in (*E*)-β-farnesene content in *CmNPR3-*RNAi plants (Fig. 5G).

**Fig. 6:**
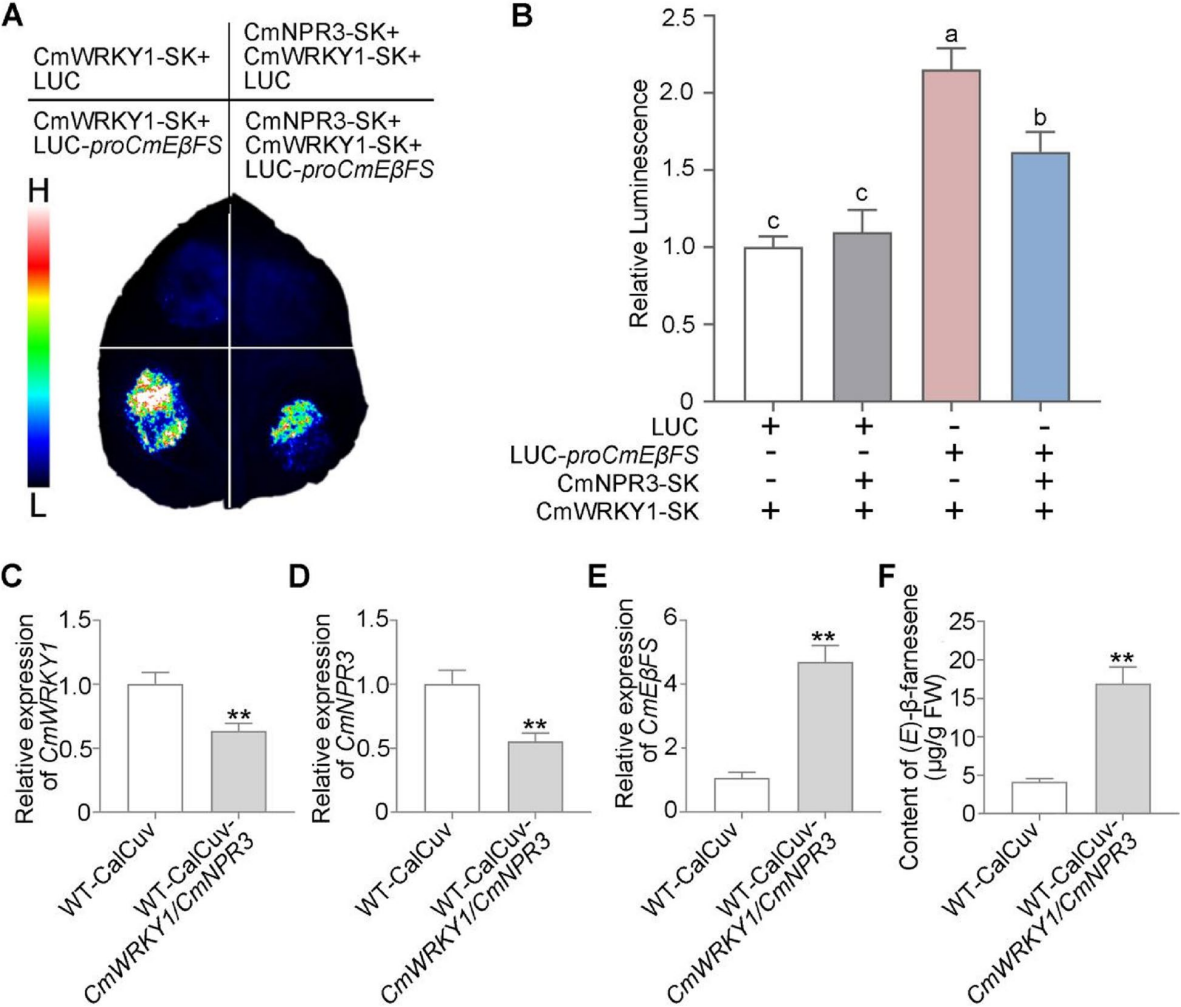
The CmWRKY1-CmNPR3 module represses the biosynthesis of (*E*)-β-farnesene by regulating the expression of *CmEβFS*. **A**-**B** Dual-luciferase (LUC) analysis showing that co-infiltration of CmWRKY1and CmNPR3 strengthens CmWRKY1-mediated transcriptional suppression of *CmEβFS* promoter in *N. benthamiana* leaves (left). Normalized LUC activities are shown as LUC/REN ratios (right). Data represent the mean ± SD of three biological replicates (*n* = 3). Statistical significance was assessed using one-way ANOVA analysis of variance (*P* < 0.05). **C**-**E** RT-qPCR analysis of *CmWRKY1*, *CmNPR3* and *CmEβFS* expression in flowers of the WT-CalCuv and WT-CalCuv-*CmWRKY1*/*CmNPR3* plants. *CmUBI* was quantified and served as the internal control. **F** GC–MS analysis of (*E*)-β-farnesene content in flowers of the WT-CalCuv and WT-CalCuv-*CmWRKY1*/*CmNPR3* plants. For RT-qPCR analysis, five flowers at stage 3 were pooled together as one biological replicate. For GC–MS analysis in **(F)**, one flower at stage 3 was collected as one biological replicate. Values are the means ± SD of three biological replicates. For (**C**)-(**F**), statistical significance was determined by Student’s *t*-test (**P < 0.01)

To further verify the function of CmWRKY1-CmNPR3 module, a transactivation assay was performed. Our results revealed that the CmWRKY1-CmNPR3 module significantly enhances CmWRKY1-mediated repression of *CmEβFS* promoter activity (Fig. 6A, B). In order to clarify the function of CmWRKY1-CmNPR3 module in chrysanthemum, we simultaneously silenced both *CmWRKY1* and *CmNPR3* in the WT plants using a modified cabbage leaf-curl geminivirus vector (CaLCuV) containing the artificial microRNA-*CmNPR3* or microRNA-*CmWRKY1*. RT-qPCR assay confirmed that the expressions of *CmNPR3* and *CmWRKY1* were significantly reduced in plants with double silencing of *CmWRKY1* and *CmNPR3* (Fig. 6 C, D). Compared to WT-CaLCuV plants, the double-silenced plants exhibited a significant reduction in the inhibitory effect of CmWRKY1 on *CmEβFS*, leading to a substantial increase in (*E*)-β-farnesene accumulation (Fig. 6E, F). These results indicated that CmWRKY1-CmNPR3 module inhibits (*E*)-β-farnesene biosynthesis by negatively regulating *CmEβFS* expression in chrysanthemum.

### SA regulates (*E*)-β-farnesene biosynthesis via the CmWRKY1-CmNPR3 module

Since CmNPR3 functions as an SA receptor, we hypothesized that SA participates in regulating (*E*)-β-farnesene biosynthesis. To test this hypothesis, we first assessed the SA levels during floral development. Our results showed a significant increase in SA levels from stage 1 to stage 2, followed by a significant decrease in stage 3. However, SA levels at stage 3 remained significantly higher than those at stage 1 (Fig. S13A). Furthermore, we found that *CmNPR3* expression gradually increased during floral development (Fig. S13B). These results indicated that the effects of SA continued to increase during floral development. Furthermore, we found that the SA levels in ray and disc florets were significantly higher than those in phyllaries and receptacles (Fig. S13C). Consistent with the distribution of SA content, the *CmNPR3* expression was significantly higher in ray and disc florets than in phyllaries and receptacles (Fig. S13D). Collectively, these results indicated that SA negatively regulates (*E*)-β-farnesene biosynthesis.

**Fig. 7:**
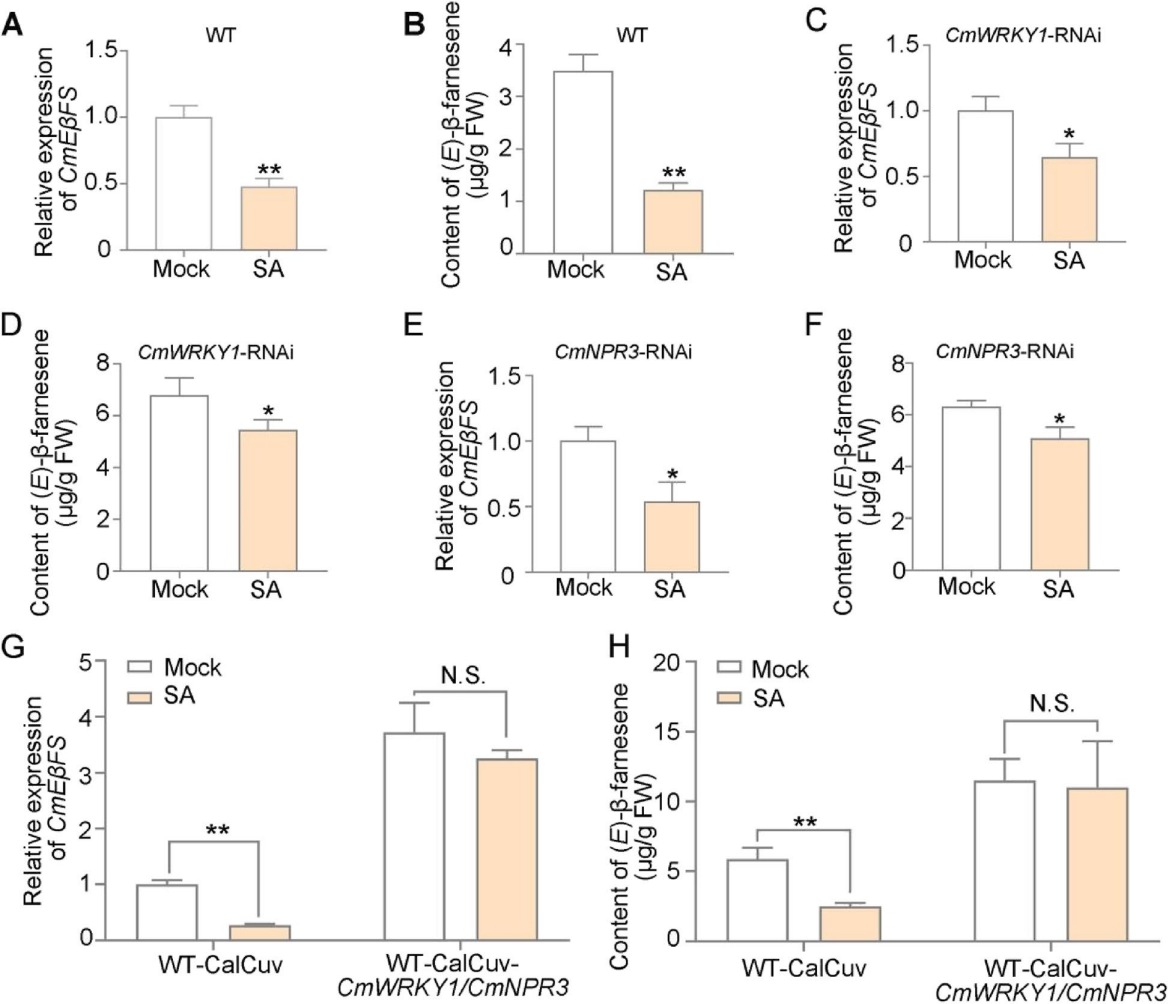
CmWRKY1 respond to SA, regulating the expression of *CmEβFS*. **A** Expression of *CmEβFS* in flowers of mock-treated and SA-treated plants. **B** (*E*)-β-farnesene content in flowers of mock-treated and SA-treated plants. **C** Expression of *CmEβFS* in flowers of *CmWRKY1*-RNAi plants and SA-treated plants. **D** (*E*)-β-farnesene content in flowers of *CmWRKY1*-RNAi plants and SA-treated plants. **E** Expression of *CmEβFS* in flowers of *CmNPR3*-RNAi plants and SA-treated plants. **F** (*E*)-β-farnesene content in flowers of *CmNPR3*-RNAi plants and SA-treated plants. **G)** Expression of *CmEβFS* in flowers of WT-CalCuv and WT-CalCuv-*CmWRKY1*/*CmNPR3* plants, and SA-treated plants. **H** (*E*)-β-farnesene content in flowers of WT-CalCuv and WT-CalCuv-*CmWRKY1*/*CmNPR3* plants, and SA-treated plants. For SA treatment, flowers at stage 1 were treated with 0.1 mM SA. For RT-qPCR analysis, five flowers were pooled together as one biological replicate. *CmUBI* was quantified and served as the internal control. For GC–MS analysis, one flower was collected as one biological replicate. Values are the means ± SD of three biological replicates (*n* = 3). Statistical significance was determined by Student’s *t*-test (*P < 0.05, **P < 0.01)

To explore the relationship between SA and (*E*)-β-farnesene biosynthesis in chrysanthemum, we treated flowers with SA and assessed *CmWRKY1*, *CmNPR3* and *CmEβFS* expressions in flowers using RT-qPCR. We also detected the (*E*)-β-farnesene content using GC–MS. SA treatment of flowers at stage 1 inhibited *CmEβFS* expression while upregulating *CmWRKY1* and *CmNPR3* expression, thereby suppressing (*E*)-β-farnesene biosynthesis (Fig. 7A, B, Fig. S13E, F). Furthermore, we treated *CmWRKY1*-RNAi, *CmNPR3*-RNAi, and WT-CaLCuV*-CmWRKY1/CmNPR3* plants with SA and assessed *CmWRKY1*, *CmNPR3* and *CmEβFS* expressions in flowers using RT-qPCR (Fig. 7, Fig. S13G-J). We also detected the (*E*)-β-farnesene content using GC–MS. As shown in Fig. 7, SA treatment of *CmWRKY1*-RNAi and *CmNPR3*-RNAi plants resulted in decreased expression of *CmEβFS* and reduced accumulation of (*E*)-β-farnesene (Fig. 7C-F). However, *CmWRKY1/CmNPR3-*CaLCuV plants did not alter *CmEβFS* expression or (*E*)-β-farnesene accumulation (Fig. 7 G-H). A comprehensive analysis of the expression patterns of *CmEβFS* and *CmWRKY1* during floral development revealed that *CmEβFS* was highly expressed at floral bud stage, likely due to the relatively low endogenous SA levels at this stage. However, with increasing SA levels during the initial flowering stage, *CmEβFS* expression significantly decreased, while *CmWRKY1* expression increased substantially. These results suggested that SA participates in (*E*)-β-farnesene biosynthesis by regulating *CmWRKY1* expression.

**Fig. 8:**
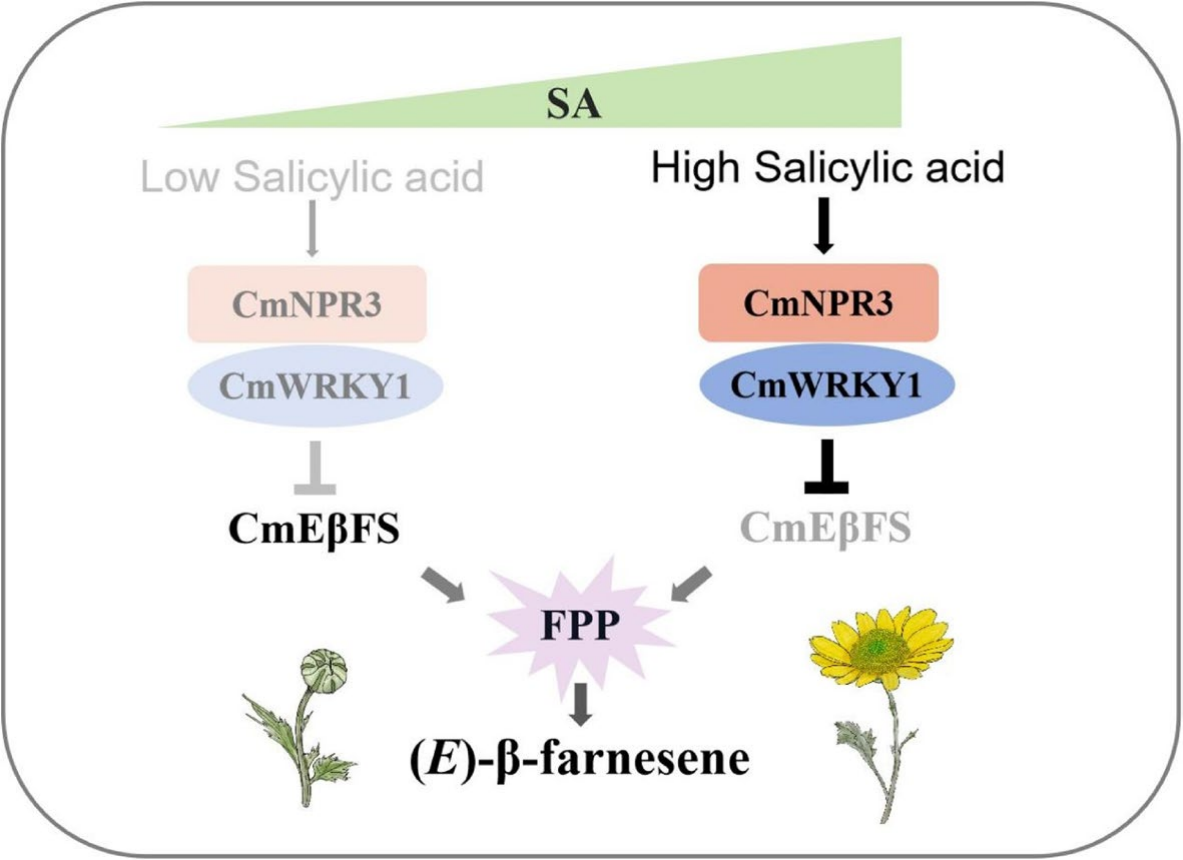
A proposed regulatory mechanism for (*E*)-β-farnesene biosynthesis in chrysanthemum. *CmEβFS*, which encodes an (*E*)-β-farnesene synthase, is highly expressed in the bud stage due to the low expression of negative regulatory factor CmWRKY1. During the flowering process, the concentration of SA gradually increases. The expression of *CmWRKY1* increases rapidly. CmWRKY1 interacts with SA receptor, CmNPR3, to form a CmWRKY1-CmNPR3 module that inhibits *CmEβFS* expression, leading to decreased (*E*)-β-farnesene biosynthesis

In summary, our findings revealed a regulatory mechanism underlying (*E*)-β-farnesene biosynthesis in chrysanthemum and that it is modulated, at least in part, by SA. We speculated that (*E*)-β-farnesene biosynthesis primarily occurs during the bud stage when the endogenous SA levels are low. During this period, the expression of negative regulatory *CmWRKY1* was low, promoting the expression of the (*E*)-β-farnesene biosynthesis enzyme-encoding gene *CmEβFS*. However, from the bud stage to the later flowering stage, SA concentration increases significantly, causing the negative regulatory module CmWRKY1-CmNPR3 to inhibit *CmEβFS* expression (Fig. 8).

## Discussion

### (*E*)-β-farnesene is a major fragrance component in fruit-scented chrysanthemum

(*E*)-β-farnesene, a biologically important sesquiterpene, is biosynthesized from farnesyl pyrophosphate (FPP) via enzymatic catalysis by EβFS. As a key volatile compound, it contributes substantially to the characteristic fragrance of various plant species, including *Polygonum hydropiper* (Miyazawa and Tamura 2007), sweet orange (Niu et al. 2024), chamomile (Umnahanant and Chickos 2024), and *Erigeron sublyratus* (An et al. 2025), typically imparting a pleasant aroma. Current research on (*E*)-β-farnesene has primarily focused on its quantitative analysis. In chrysanthemum, (*E*)-β-farnesene not only contributes to floral aroma but also functions as the dominant constituent of natural aphid alarm pheromones. Its emission acts as a dual defense mechanism, deterring aphid infestation while attracting natural predators for indirect protection (Li et al. 2019, 2024b). However, its biosynthetic mechanism requires further investigation. Our previous study found that (*E*)-β-farnesene was highly abundant in fruit-scented chrysanthemums (Wang et al. 2023). In this study, we have elucidated the biosynthetic pathway and regulatory mechanisms underlying (*E*)-β-farnesene production during floral development in chrysanthemum.

The biosynthesis and emission dynamics of VOCs in flowers are developmentally regulated, exhibiting distinct species-specific temporal patterns. Previous studies have demonstrated distinct VOCs emission profiles during floral development across different species. In *Freesia*, VOCs emissions progressively increase, reaching maximum levels at full flower opening (Gao et al. 2018). In contrast, *Rosa gigantea* demonstrates a biphasic emission pattern, characterized by upregulation from young buds to initial flowering stages, followed by a gradual decline from half-open to fully open flowers (Zhou et al. 2024). These differential emission patterns highlight the species-specific regulatory mechanisms underlying VOCs biosynthesis and emission throughout floral development.

To precisely characterize VOCs dynamics during chrysanthemum floral development, we defined full-open flower stage by three distinct morphological characteristics: fully expanded ray florets, completely opened outer disc florets, and partially opened second outer disc florets. Consistent with findings in *Freesia*, *(E*)-β-farnesene accumulation in chrysanthemum progressively increased during floral development (Fig. 1E). Importantly, (*E*)-β-farnesene remained the most abundant terpenoid compound throughout all developmental stages (Fig. 1D), suggesting its fundamental role in chrysanthemum floral biology.

Phyllaries and receptacles are the primary organs where sesquiterpene accumulate in chrysanthemum (Zhang et al. 2021; 2024). Similarly, our findings indicated that the major component (*E*)-β-farnesene predominantly accumulated in the phyllaries and receptacles (Fig. 1F, Fig. S3). Additionally, glandular trichomes on epidermal cells are the primary sites for terpenoid biosynthesis and release in plants (Schuurink and Tissier 2020; Guan et al. 2022; 2024). Previous studies indicate that glandular trichomes involved in volatile biosynthesis typically contain specialized subcellular compartments for liquid-phase metabolite storage, with these metabolites being released primarily upon trichome damage (Hammond and Mahlberg 1977; Jorge and Jules 1995; Schuurink and Tissier 2020). Here, we observed that the glandular trichomes were disrupted, and the sheaths were collapsed during stage 3 (Fig. 1C). This finding was consistent with (*E*)-β-farnesene levels during floral development (Fig. 1D), suggesting that the highest (*E*)-β-farnesene secretion occurs during the later stages of floral development. Combined with the results of glandular observations and VOCs content detection, we proposed that the biosynthesis of VOCs occurs in the early stage of floral development, while volatile release is primarily regulated during the later stages.

### *CmEβFS* is negatively regulated by CmWRKY1 during floral development

The biosynthesis of VOCs is primarily regulated by the expression levels of enzyme-encoding genes and enzyme activities of key biosynthetic proteins (Bao et al. 2023; Li et al. 2025). Terpenoids are known to be synthesized through the catalytic action of the corresponding terpene synthase (Dudareva et al. 2013; Li et al. 2024a). EβFS catalyzes the conversion of FPP to (*E*)-β-farnesene in many plants (Wang et al. 2019; Zhang et al. 2024). In this study, we found that CmEβFS catalyzed (*E*)-β-farnesene production in chrysanthemum (Fig. 3). Although transcriptome sequencing revealed significantly higher expression levels of *Unigene1507_All* and *CL7457.Contig2_All* compared to *CmEβFS* (Fig. S5), their protein-catalyzed products either failed to produce detectable (*E*)-β-farnesene or generated a primary product that was not a major aroma compound in chrysanthemums (Fig. S14). This discrepancy suggests potential inefficiencies in translation or post-translational modifications of these genes, ultimately impairing their catalytic activity. Other than the detection of *EβFS* expression in plants, the molecular regulatory mechanism of *EβFS* remains elusive.

The WRKY transcription factor family is widely recognized for its pivotal roles in regulating plant growth, defense and development (Song et al. 2023b). Previous studies have demonstrated that WRKY transcription factors are involved in regulating plant resistance to aphids. In chrysanthemum, WRKY48 enhances aphid resistance, while WRKY53 negatively regulates this defense mechanism (Li et al. 2015; Zhang et al. 2020). Several studies have demonstrated that WRKY1 positively regulates terpenoid biosynthesis. In *Catharanthus roseus*, WRKY1 positively regulates the terpenoid indole alkaloid biosynthesis (Suttipanta et al. 2011). In *Artemisia annua*, WRKY1 increased the content of artemisinin by regulating the expression of the key enzyme genes, *ADS* and *CYP*, in artemisinin biosynthetic pathway (Jiang et al. 2016). In this study, we demonstrated that CmWRKY1 regulates aromatic compound biosynthesis in chrysanthemums. Specifically, we revealed that CmWRKY1 functions as a negative regulator of *CmEβFS*, inhibiting (*E*)-β-farnesene synthesis in chrysanthemum (Fig. 4). However, the upstream transcriptional regulation mechanisms of CmWRKY1 remains unclear, and its potential involvement in chrysanthemum aphid resistance requires further investigation.

### SA signaling mediates (*E*)-β-farnesene biosynthesis via CmNPR3 during floral development

SA acts as a pivotal phytohormone involved in diverse metabolic processes in plants, including the regulation of VOCs biosynthesis (Yue et al. 2023). It has been reported that a concentration-dependent regulatory role of SA in modulating terpenoids biosynthesis across different plant species. In grapes, optimal SA concentration (50 mg/L) significantly enhanced monoterpene accumulation, whereas a higher concentration (200 mg/L) did not have a significant inhibitory effect (Yue et al. 2023). Similarly, in citrus, SA treatment at 10 μM downregulated *CsTPS21*, a key gene encoding β-ocimene synthase (Bin et al. 2023). These findings suggest that SA-mediated regulation of volatile biosynthesis is both species-specific and concentration-dependent, although the precise molecular mechanism remains unclear. Consistent with findings in other species studies, SA also functions to regulate terpenoid biosynthesis in chrysanthemum. Our study revealed an inverse relationship between endogenous SA levels and (*E*)-β-farnesene content during floral development, implying that an increase in SA levels may inhibit (*E*)-β-farnesene biosynthesis. Moreover, the exogenous SA (0.1 mM) treatment effectively downregulated *CmEβFS* expression, resulting in a decrease in the biosynthesis of (*E*)-β-farnesene, suggesting a conserved regulatory role of SA in terpenoid metabolism across diverse plant species (Fig. 7).

SA-mediated signaling transduction in plants requires the involvement of receptors and TFs (Jia et al. 2023). NPR1 is a key receptor of the SA signaling pathway, and NPR3 and NPR4 are homologous to NPR1, exhibit redundant functions (Fu al. 2012; Ding et al. 2018). NPRs do not bind directly to the promoters of downstream genes; instead, other TFs are necessary as binding partners. Several TFs, including TGAs, WRKYs, and NIM1-interacting proteins (NIMINs), play significant roles in the SA signaling pathway (Peng et al. 2021). NPR3 and NPR4 function as transcriptional co-repressors inhibited by SA to modulate downstream gene expression (Ding et al. 2018). However, the involvement of NPRs in chrysanthemum aroma biosynthesis remains poorly understood. Here, we demonstrate that CmNPR3 physically interacts with CmWRKY1 (Fig. 5), thereby potentiating CmWRKY1-mediated repression of *CmEβFS* expression. Consistent with this regulatory module, double silencing of *CmNPR3* and *CmWRKY1* abolished SA responsiveness, resulting in unaltered (*E*)-β-farnesene biosynthesis upon SA treatment (Fig. 7). Given that SA is a key defense signaling molecule in plants and (*E*)-β-farnesene serves as both a critical aphid alarm pheromone and a floral volatile, we hypothesize that SA-mediated regulation of (*E*)-β-farnesene biosynthesis through the CmNPR3-CmWRKY1 module may confer dual ecological benefits to chrysanthemums: enhanced aphid resistance and potential modulation of pollinator attraction. This potential mechanism remains to be elucidated in the future. In summary, our findings revealed that SA regulates of (*E*)-β-farnesene biosynthesis through a receptor-mediated signaling pathway. CmNPR3 interacted with CmWRKY1 to form a CmNPR3-CmWRKY1 module, that mediates SA-dependent regulation of (*E*)-β-farnesene biosynthesis during floral development (Fig. 8).

## Materials and Methods

### Plant materials and growth conditions

The chrysanthemum (*Chrysanthemum* × *morifolium*) cultivar ‘Xiaokuixiang’ was used in this study. The floral development was categorized into three stages: the bud stage (stage 1), the initial flower stage (stage 2), and the blooming stage (stage 3). The flower divided into four organs: ray floret, disc floret, phyllary and receptacle. The 45-day-old plants were transplanted into 9-cm-diameter pots filled with a peat: vermiculite (1:1, v/v) mixture and grown in a culture room at 22 ± 2 °C with 40% relative humidity and 300 μmol m^−2^ s^−1^ illumination. Plants were grown for 2 months under a 16-h/8-h (light/dark) photoperiod and then switched to an 8-h/16-h light/dark photoperiod. *Nicotiana benthamiana* plants were grown in 9-cm-diameter pots filled with a peat: vermiculite (1:1, v/v) mixture in a culture room at 22 ± 2℃ with 50%–55% relative humidity, 100 μmol m^−2^ s^−1^ illumination, and a 16-h/8-h (light/dark) photoperiod. Plants were grown for approximately 4 weeks for experiments.

### Identification of volatiles using solid-phase microextraction gas chromatography–mass spectrometry

Chrysanthemum flower was cut and immediately placed in sampling bottles (VAAP-320018EM-2375–100, ANPEL Laboratory Technologies, Shanghai, Inc, Shanghai), and 15 µL of internal standard (43.25 ng·μL^−1^ ethyl decanoate, Shanghai Aladdin Biochemical Technology Co., Ltd., Shanghai) was added to each bottle. Quantification was achieved using ethyl decanoate standard. For each experiment, three biological repeats were performed. The sample was placed into a sampling bottle in a 45 °C water bath, the extraction head was inserted, and VOCs were extracted from the headspace for 20 min. Gas chromatography–mass spectrometry (GC–MS) analysis of the headspace material was conducted using an Agilent 7890B/7200 mass spectrometer. The chromatographic conditions were as follows: injection port temperature, 250 °C; injection mode, split flow; total flow rate, 27.4 mL/min; split ratio, 20; ion source temperature, 200 °C; and interface temperature, 250 °C. The total analysis time was 30 min. The initial temperature of 40 °C was maintained for 1 min and then raised to 280 °C at a rate of 10 °C/min and maintained for 5 min. The solvent delay time was 2.5 min. Mass spectrum conditions were as follows: detector, 1 kV; mass scanning range 30–500 m/*z*; full scanning mode. Volatiles were identified by comparing the mass spectra and retention times with the NIST17 mass spectra library and standard samples.

### Transcriptomic sequencing analysis

Total RNA was extracted from the chrysanthemum flowers at stages 1 and 2, respectively, followed by library construction, RNA sequencing, and data analysis according to the protocols described previously (Wei et al. 2017). Ten flowers from each developmental stage were pooled together as one biological replicate. Three biological replicates were analyzed in this experiment. A total of 38.48 Gb of data was measured using the BGISEQ-500 platform of BGI Genomics Co., Ltd. (Shenzhen, China). After assembly and redundancy removal, 166,324 Unigenes were obtained, with a total length, average length, N50, and GC content of 188,673,266 bp, 1,134 bp, 1,697 bp, and 39.24%, respectively (Table S1−3). De novo assembly of high-quality clean reads into contigs was performed using the Trinity program (Zhong et al. 2011).

### RNA extraction and RT-qPCR analysis

Total RNA extraction using RNAiso Plus reagent (TaKaRa, Japan) according to the manufacturer’s instructions. cDNAs were synthesized from 1 μg of total RNA using the M5 Super plus RT-qPCR kit with gDNA Remover (Mei5bio, Beijing, China). RT-qPCR reactions (20 μL volume containing 1 μL cDNA as the template) were run using the StepOne Real-Time PCR System (Applied Biosystems) in standard mode with the M5 HiPer One-Step RT-qPCR kit (SYBR Green) (Mei5bio, Beijing, China). *CmUBI* (*UBIQUITIN*; GenBank accession: NM_112764) was used as the internal reference gene (Lyu et al. 2022). The relative expression of genes was calculated using the 2^−ΔΔCT^ method (Livak and Schmittgen 2001). Gene-specific primers are listed in Table S8.

### Gland observation

To visually characterize the glands, ray florets, disc florets, phyllaries, and receptacles were dissected from different floral stages of chrysanthemum, including stage 1, stage 2, and stage 3. Immediately after dissection, the samples were observed by scanning electron microscopy (Hitachi TM4000, Japan) with an accelerating voltage of 15 kV.

### Separation of glands and determination of volatiles in glands

The receptacles and phyllaries of chrysanthemum were placed into 2 mL centrifuge tubes, rapidly cooled in liquid nitrogen for 5 s, and vortexed oscillation for 5 s, alternating 15 times. After oscillation, the sample was filtered into a centrifuge tube through a 30 μm nylon mesh. Next, 0.5 mL of chloroform was added, and the tube was covered with a lid and shaken continuously for 5 min and filtered into a glass bottle (VAAP-31509-1232 A-100, ANPEL Laboratory Technologies (Shanghai) Inc, Shanghai) using an organic-phase needle filter and analyzed by GC–MS.

### Preparation of recombinant proteins

Full-length ORF for *CmEβFS*, 1–606 bp ORF for *CmWRKY1* were cloned into the *pEasy*®-Blunt E1 vector and transformed into expression strain *Escherichia coli* DE3 (Beijing Genes and Biotech Co., Ltd., China) to produce recombinant His-CmEβFS and His-CmWRKY1. Full-length cDNA for *CmNPR3* were cloned into the pGEX-4 T-2 vector and transformed into expression strain *Escherichia coli* DE3 (Beijing Genes and Biotech Co., Ltd., China) to produce recombinant GST-CmNPR3. Recombinant proteins were induced with 0.2 mM isopropylthio-β-galactoside (IPTG) at 16℃ overnight. His-tagged (CmEβFS-His and CmWRKY1-His) were purified using Ni Sepharose™ 6 Fast Flow (GE Healthcare Bio-Sciences ABSE-751 84 Uppsala, Sweden), GST-tagged (GST and CmNPR3-GST) were purified using Glutathione Sepharose™ 4B (GE Healthcare Bio-Sciences ABSE-751 84 Uppsala, Sweden). The purified recombinant proteins were used in antibody generation for CmEβFS, enzymatic assays in vitro (CmEβFS), pull-down MS (CmWRKY1 recombinant proteins) and pull-down experiment.

### Antibody generation and specificity validation

For the generation of the CmEβFS antibody, recombinant proteins identified by mass spectrometry (Biological mass spectrometry laboratory, College of Biological Sciences, China Agricultural University, Beijing, China) were used as an immunogen to produce polyclonal antiserum in rabbits (Institute of Genetics and Developmental Biology, Chinese Academy of Sciences, Beijing, China). To validate the specificity and functionality of CmEβFS antibody, the total proteins of chrysanthemum flowers at stage1 was extracted and subjected to immunoblotting. Purified His-CmEβFS recombinant proteins was used to be a positive control (Fig. S7).

### Protein extraction and immunoblot analysis

Total proteins from chrysanthemum flowers at stage 1 (bud stage) was extracted in a plant protein extraction solution (Catalog #HX18612, Beijing Huaxing Bochuang Gene Technology Co., Ltd., Beijing, China). The samples were ground to powder in liquid nitrogen, then added extraction solution buffer and vortexed oscillation thoroughly mixed. After centrifugation at 12,000 g for 15 min at 4℃ and then collect the supernatants. The samples were separated by 12% sodium dodecyl sulfate polyacrylamide gel electrophoresis (SDS-PAGE) and analyzed by immunoblot using anti-His (1:1,000, Beyotime Biotechnology, Shanghai, China) and anti-CmEβFS (1:1,000) antibodies.

### Enzymatic assays in vitro and in vivo

For in vivo assays, total flower proteins were extracted from stage 1. Total proteins were incubated with an anti-CmEβFS antibody at 4 °C for 12 h, and then 20 µL of BeyMag Protein A + G magnetic beads (Beyotime Biotechnology, Shanghai, China) was added at 4 °C for 8 h, and the beads were washed five times with 1 mL 1 × TBS. The CmEβFS activity assays were conducted as described (Gao et al. 2018) with some modifications. The reaction mixture for the enzyme assay consisted of the purified proteins (for in vitro) or beads with CmEβFS proteins (in vivo), 20 mM DTT, 100 mM MgCl_2_, 25 mM HEPES (pH 7.5), and 2.5% glycerol for 1 h at 37℃, and the products were detected by GC–MS as described above.

### Phylogenetic analysis

Phylogenetic analyses were performed using MEGA version 10 and the neighbor-joining method with bootstrap analysis (1000 replicates) (Tamura et al. 2011). The accession IDs of amino acid sequences used for phylogenetic analysis and alignments are listed in Table S6, 7.

### Stable transformation of chrysanthemum plants

To construct RNAi vectors, 425-bp sense-specific fragments using *Asc*I/*Swa*I and antisense-specific fragments using *Bam*HI/*Pac*I sites of *CmEβFS*, *CmWRKY1* and *CmNPR3* were cloned into pFGC1008. The contructs were introduced into *Agrobacterium tumefaciens* strain EHA105 (Beijing Tsingke Biotech Co., Ltd., Beijing) and transformed into chrysanthemum by Agrobacterium-mediated transformation (Hong et al. 2006). The primers used for vector construction are listed in Table S8.

### Subcellular localization of proteins

To construct a vector for determining subcellular localizations, the *CmEβFS*, *CmWRKY1* and *CmNPR3* ORF sequences (without terminators) were fused with GFP in pCAMBIA-1300 (Huang et al. 2022) using the *Xba*I/*Kpn*I sites. The constructs were transformed into *Agrobacterium tumefaciens* strain GV3101 (Beijing Tsingke Biotech Co., Ltd., Beijing) and then introduced into *N. benthamiana* leaves using a needle-less syringe (Wei et al. 2017). p35S-H2B-mCherry was used as a nuclear localization marker, and p35S-GFP was used as a control. Fluorescence signals were observed under a Nikon A1 confocal laser scanning microscope (Nikon, Japan). The GFP and mCherry signals were excited with 488 and 561 nm, respectively. The primers used for vector construction are listed in Table S8.

### Promoter cloning

The promoter reference sequence of *CmEβFS* was obtained from the chrysanthemum reference genome (Song et al. 2023a). Specific primers were then designed by NCBI. PCR reactions were performed using genomic DNA as templates. PCR products of appropriate lengths were cloned into the One-step ZTOPO-Blunt/TA (ZOMANBIO) and then transformed into Trelief 5α Chemically Competent Cells (Tsingke) before sequencing. Primer sequences are listed in Table S8.

### Yeast one-hybrid assay

To construct the plasmids used in yeast one-hybrid assays, the promoter sequence of *CmEβFS* was inserted into the pAbAi vector (Clontech, Japan) using *Kpn*I/*Xho*I sites. The *CmWRKY1* ORF were inserted into pGADT7 using *Eco*RI/*Bam*HI sites. The Matchmaker Gold Yeast Single Hybrid Library Screening System (Clontech, Japan) was used to test for protein–DNA interactions in yeast cells. A one-hybrid library using high-quality chrysanthemum flower cDNA was constructed to identify genes that act upstream of *CmEβFS*. Interaction assays were performed using 200 ng/mL aureobasidin A (AbA) (Clontech, Japan) under strict selection on synthetic dropout (SD)/− Ura/− Leu medium. Primer sequences are listed in Table S8.

### ChIP-qPCR analysis

CmWRKY1-GFP, and an empty vector control were introduced into *A. tumefaciens* strain GV3101 (Wang et al. 2022). ChIP experiments were performed according to a general protocol described previously (Zhang et al. 2021; Wang et al. 2022) with some modification. Two grams of fresh leaves was ground to powder in liquid nitrogen, suspended in extraction buffer (0.4 M sucrose, 10 mM Tris‐HCl, pH 8.0, 10 mM MgCl_2_), and crosslinked for 5 min in 1% (v/v) formaldehyde. The chromatin mixture was separated and then fragmented by sonication using a sonifier (Branson S‐250D). The solubilized chromatin was incubated with 10 μL anti‐GFP antibody (BE2001; Easybio, Beijing) and 20 μL BeyoMag™ Protein A + G Magnetic Beads (Beyotime Biotechnology, Shanghai) for 8 h. After washing, the eluted samples were incubated at 65 °C for 6 h to reverse the crosslinking. The co‐precipitated DNA was purified with a QIAquick PCR Purification Kit (Qiagen GmbH, Germany) and analyzed by qPCR. Primer sequences are listed in Table S8.

### Dual-luciferase reporter assay in *N. benthamiana*

The recombinant vectors (the *CmEβFS* promoter sequence was inserted pGreenII 0800-Luc using *Hind*III/*Bam*HI sites, and the ORF of *CmWRKY1* and *CmNPR3* were inserted into pGreenII 62-SK using *Eco*RI/*Kpn*I sites) were introduced into *Agrobacterium tumefaciens* strain GV3101 containing pMP90 and pSoup plasmids (Hellens et al. 2000). A mixture of *A. tumefaciens* cultures was infiltrated into *N. benthamiana* leaves as previously described (Wei et al. 2017). The activities of LUC and REN were measured using a dual-luciferase reporter reagent (Promega, USA) and a GloMax 20/20 luminometer (Promega, USA). LUC images were captured using an iKon-L936 imaging system (Andor Tech, Belfast, UK). Primer sequences are listed in Table S8.

### Pull down mass spectrometry

Total proteins were extracted from chrysanthemum at stage 1 using a plant protein extraction solution (Catalog #HX18612, Beijing Huaxing Bochuang Gene Technology Co., Ltd., Beijing, China). Recombinant His-CmWRKY1 was individually incubated with total proteins, anti-His antibody (Beyotime Biotechnology, Shanghai) was added for incubating overnight at 4 °C. Then 20 µL of BeyMag Protein A + G magnetic beads (Beyotime Biotechnology, Shanghai) was added at 4 °C for 8 h. Beads with protein were washed 5 times with 1 × TBS. After SDS–PAGE, the gel was analyzed by LC–MS/MS (Biological mass spectrometry laboratory, College of Biological Sciences, China Agricultural University, Beijing, China). Primer sequences are listed in Table S8.

### Yeast two-hybrid assays

Yeast two-hybrid assays were performed using the Matchmaker GAL4 two-hybrid system (Clontech, Shiga-ken, Japan). The ORF of *CmNPR3* was inserted into the pGADT7 vector using *Eco*RI/*Bam*HI sites (Chien et al. 1991). The ORF of *CmWRKY1* was inserted into pGBKT7 using *Eco*RI/*Sal*I sites (Louvet et al. 1997). The recombinant plasmids were co-transformed into yeast strain Y2HGold. pGADT7 or pGBKT7 empty vector as negative control. Transformants were grown on SD/–Trp–Leu plates and transferred to SD/–Trp/–Leu/–His/–Ade plates for growth analysis. Primer sequences are listed in Table S8.

### BiFC assay

To construct a vector for bimolecular fluorescence complementation (BiFC), the ORF without terminators of *CmNPR3* was inserted into 35S-SPYCE(M) using *Xba*I/*Kpn*I sites, and the ORF without terminators of *CmWRKY1* was inserted into 35S-SPYNE(R)173 using *Xba*I/*Kpn*I sites. Recombinant vectors or control vectors were transferred into *Agrobacterium tumefaciens* strain GV3101. Mixtures of *A. tumefaciens* cultures expressing either 35S-SPYCE(M) or 35S-SPYNE(R) (v:v, 1:1) were infiltrated into *N. benthamiana* leaves using a needle-less syringe (Wei et al. 2017). After 3 days, yellow fluorescent protein was imaged using a Nikon A1 confocal laser scanning microscope (Nikon, Japan). YFP signal with excitation at 488 nm. Primer sequences are listed in Table S8.

### Pull-down assay

Recombinant proteins CmWRKY1-His were incubated with GST or CmNPR3-GST bound to Glutathione Sepharose™ 4B (GE Healthcare Bio-Sciences ABSE-751 84 Uppsala, Sweden) overnight at 4℃ in lysis buffer (50 mM Na_2_HPO_4_; 300 mM NaCl; 1 mM PMSF; pH 8.0). The mixture were washed with 1 × PBS (10 mM Na_2_HPO_4_; 1.8 mM KH_2_PO_4_; 140 mM NaCl; 2.7 mM KCl;) for five times. Next, add the SDS-PAGE Sample Loading Buffer (P0015B, Beyotime Biotechnology, Shanghai, China) and heated at 95℃ for 10 min. The products were detected using western blots with anti-His (AF2876, Beyotime Biotechnology, Shanghai, China) and anti-GST (AF2888, Beyotime Biotechnology, Shanghai, China) antibodies.

### Co-immunoprecipitation (Co-IP) assay

Four-week-old *N. benthamiana* leaves were co-infiltrated with *Agrobacterium tumefaciens* carrying the plasmid *Super*_*pro*_*−3* × *Flag*-CmWRKY1 or *Super*_*pro*_*−3* × *Flag*-GFP and *Super*_*pro*_-*6* × *Myc*-CmNPR3. The *N. benthamiana* leaves were collected for Co-IP assays after three days. The total proteins were extracted with a plant protein extraction solution (Catalog #HX18612, Beijing Huaxing Bochuang Gene Technology Co., Ltd., Beijing, China). The extracts were incubated with Anti-Flag Nanobody Magarose Beads (KTSM1338, AlpalifeB Inc., Shenzhen, China) for 3 h at 4℃ and washed with dilution buffer (50 mM Tris–HCl pH 7.5; 150 mM NaCl; 1 mM EDTA) five times. The beads were mixed with SDS-PAGE Sample Loading Buffer (P0015B, Beyotime Biotechnology, Shanghai, China) and heated at 95℃ for 10 min. The IP products were detected using western blots with anti-Flag (AF0036, Beyotime Biotechnology, Shanghai, China) and anti-Myc (AF0033, Beyotime Biotechnology, Shanghai, China) antibodies.

### Virus-induced gene silencing

To silence *CmWRKY1* and *CmNPR3* together in chrysanthemum, a previously reported virus-based microRNA expression system was used (Tang et al. 2010; Xu et al. 2020). A modified CaLCuV vector containing pre-cmo-*CmWRKY1*/*CmNPR3* (CaLCuV + *CmWRKY1*/*CmNPR3*) was generated and introduced into the *A.* tumefaciens strain GV3101. Sixty-day-old WT plants were immersed in infiltration buffer. The silenced plants were validated by RT-qPCR to detect the expression of *CmWRKY1* and *CmNPR3*. Three independent experiments were performed, and at least 6 positive plantlets were used to observe the phenotypes (Zhao et al. 2023). Primer sequences are listed in Table S8.

### Determination of SA content

SA content was determined by Zoonbio Biotechnology Co., Ltd. (Nanjing, China) using an HPLC–electrospray ionization–tandem mass spectrometry platform with stage 1 to 3 chrysanthemum flowers and four organs (ray florets, disc florets, phyllaries and receptacles) with stage 1. Each stage was determined for three biological replicates, each consisting of 12 flowers.

### SA treatments

Chrysanthemum flowers from stage 1 to stage 3 were sprayed with 0.1 mM SA dissolved in distilled water. Distilled water was used as control. The flowers were collected after 4 h treatment for VOCs detection and gene expression analysis.

### Statistical analysis

Data are presented as the mean ± standard deviation (SD). Statistical analysis was performed with GraphPad Prism 8 software. Comparisons between two groups of data were calculated by Student’s *t*-test with two-sided (*P < 0.05; **P < 0.01). One-way ANOVA with Turkey comparisons test was used for single factor multiple comparisons. Two-way ANOVA with Turkey comparisons test was used for two factors multiple comparisons. A value of *P* < 0.05 was considered to be statistically significant.

## Supplementary Information

Supplementary Material 1: Supplementary Fig. S1. Statistical analysis of glandular trichomes development status at different development stages of chrysanthemum. Supplementary Fig. S2. Analysis of terpenoid content during floral development in chrysanthemum. Supplementary Fig. S3. Analysis of volatile terpenoid content in different floral organs. Supplementary Fig. S4. Detection of VOCs in glands. Supplementary Fig. S5. Transcriptomic analyses of terpene synthase genes. Supplementary Fig. S6. Tissue-specific expression analysis of (*E*)-β-farnesene synthase genes in chrysanthemum. Supplementary Fig. S7. Characterization of CmEβFS. Supplementary Fig. S8. Analysis of CmEβFS antibody specificity determination. Supplementary Fig. S9. Expression profiles of candidate (*E*)-β-farnesene synthase genes. Supplementary Fig. S10. Interaction analysis between candidate transcription factors and the *CmEβFS* promoter. Supplementary Fig. S11. Characterization of CmWRKY1. Supplementary Fig. S12. Characterization of CmNPR3. Supplementary Fig. S13. The effect of SA on the biosynthesis of (*E*)-β-farnesene in chrysanthemum. Supplementary Fig. S14. Identification of enzyme activity products of Unigene1507_All and CL7457.Contig2_All.

Supplementary Material 2: Supplementary Table S1. Basic information analysis of transcriptome sequencing. Supplementary Table S2. Information assembled from transcriptome sequencing data. Supplementary Table S3. Seven major functional databases for unigenes annotation. Supplementary Table S4. Candidate transcription factors interacting with *CmEβFS* promoter. Supplementary Table S5. Analysis of CmWRKY1-interacting proteins. Supplementary Table S6. Proteins used for phylogenetic tree analysis. Supplementary Table S7. CmWRKY1 homologous proteins used for phylogenetic tree analysis. Supplementary Table S8. Primers used in the study.

## Data Availability

All data supporting the findings of this study are included in the manuscript and its supplementary information.

## Abbreviations

VOCs: Volatile organic compounds
IPP: Isopentenyl diphosphate
DMAPP: Dimethylallyl diphosphate
TPSs: Terpene synthases
GPP: Geranyl diphosphate
FPP: Farnesyl diphosphate
TFs: Transcription factors
EβFS: (*E*)-β-Farnesene synthase
SA: Salicylic acid
GC-MS: Gas chromatography-mass spectrometry
Y1H: Yeast one-hybrid
ChIP-qPCR: Chromatin immunoprecipitation-quantitative polymerase chain reaction
LUC: Dual-luciferase
Y2H: Yeast two-hybrid
BiFC: Bimolecular fluorescence complementation
YFP: Yellow fluorescent protein
CaLCuV: Cabbage leaf-curl geminivirus vector
