# Supplementary material for: Salicylic acid regulates biosynthesis of floral fragrance (E)-β-farnesene via NPR3-WRKY1 module in chrysanthemum
Source: Mol Hortic. 2025 Sep 5;5:52. doi: 10.1186/s43897-025-00174-y (PMC12412252; doi:10.1186/s43897-025-00174-y)
Supplement: Supplementary file 1 — Supplementary Material 1: Supplementary Fig. S1. Statistical analysis of glandular trichomes development status at different development stages of chrysanthemum. Supplementary Fig. S2. Analysis of terpenoid content during floral development in chrysanthemum. Supplementary Fig. S3. Analysis of volatile terpenoid content in different floral organs. Supplementary Fig. S4. Detection of VOCs in glands. Supplementary Fig. S5. Transcriptomic analyses of terpene synthase genes. Supplementary Fig. S6. Tissue-specific expression analysis of (E)-β-farnesene synthase genes in chrysanthemum. Supplementary Fig. S7. Characterization of CmEβFS. Supplementary Fig. S8. Analysis of CmEβFS antibody specificity determination. Supplementary Fig. S9. Expression profiles of candidate (E)-β-farnesene synthase genes. Supplementary Fig. S10. Interaction analysis between candidate transcription factors and the CmEβFS promoter. Supplementary Fig. S11. Characterization of CmWRKY1. Supplementary Fig. S12. Characterization of CmNPR3. Supplementary Fig. S13. The effect of SA on the biosynthesis of (E)-β-farnesene in chrysanthemum. Supplementary Fig. S14. Identification of enzyme activity products of Unigene1507_All and CL7457.Contig2_All. [file 43897_2025_174_MOESM1_ESM.docx]

**Supplementary Data**


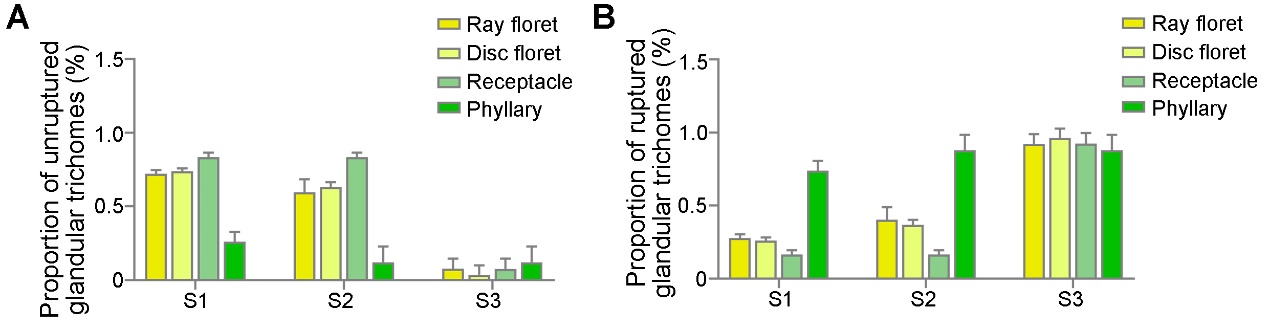


**Supplementary Fig. S1. Statistical analysis of glandular trichomes development status at different development stages of chrysanthemum**

**A)** Statistics of unruptured glandular trichomes in different flower organs during floral development (stage 1 to stage 3). **B)** Statistics of ruptured glandular trichomes in different flower organs during floral development (stage 1 to stage 3). Each flower organ counts 3 fields of view, and the field of view size for ray florets and disc florets is 1,000 × 750 μm, the field of view size for receptacles is 250 × 200 μm, the field of view size for phyllaries is 2,000 × 1,500 μm.

**
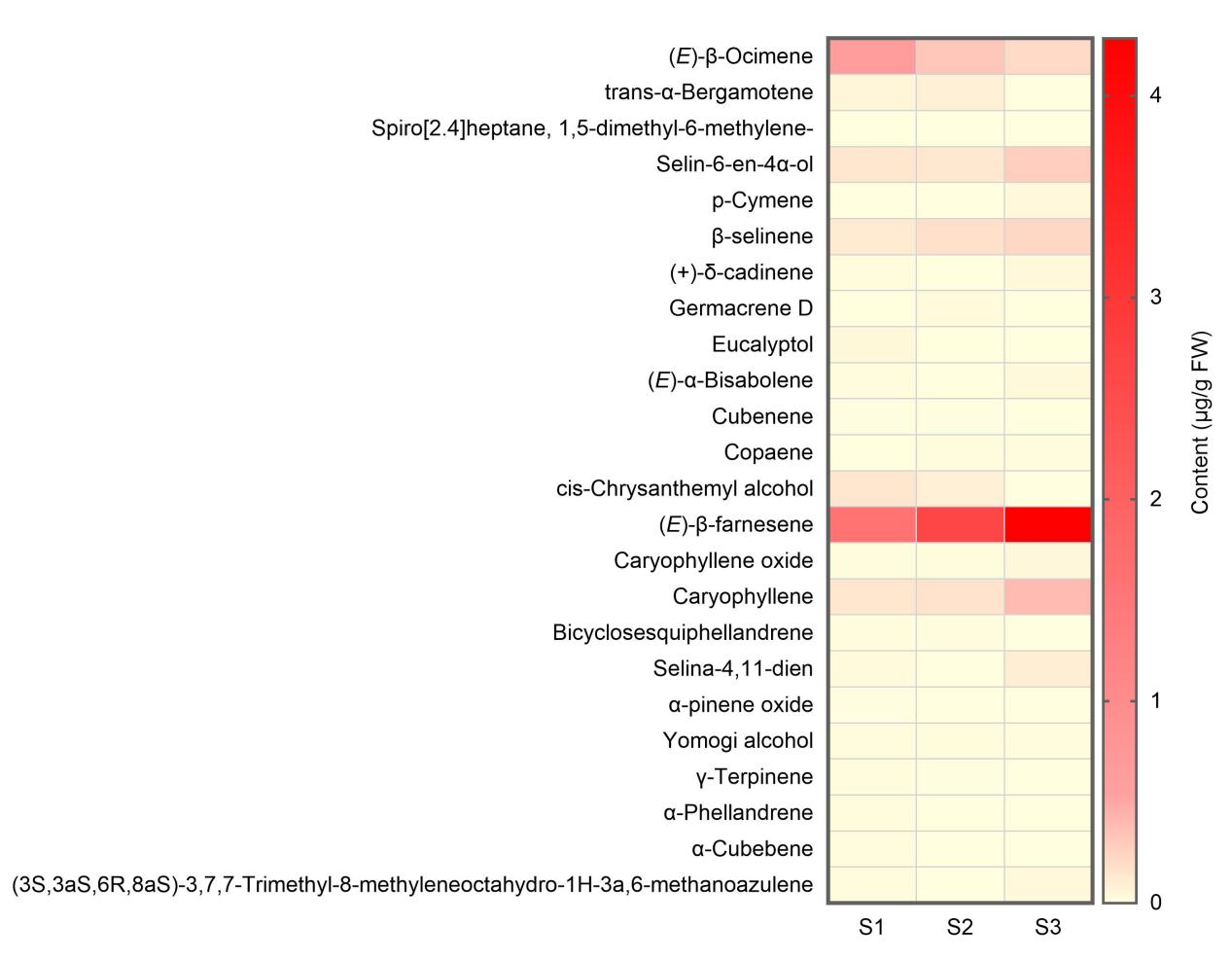
**

**Supplementary Fig. S2. Analysis of terpenoid content during floral development in chrysanthemum.** The heatmap shows the changes in the content of terpenoid detected during the three developmental stages determined by GC-MS. The color scale was used to indicate the content of the VOCs (μg/g FW). Each flower is one biological replicate; three biological replicates were performed.


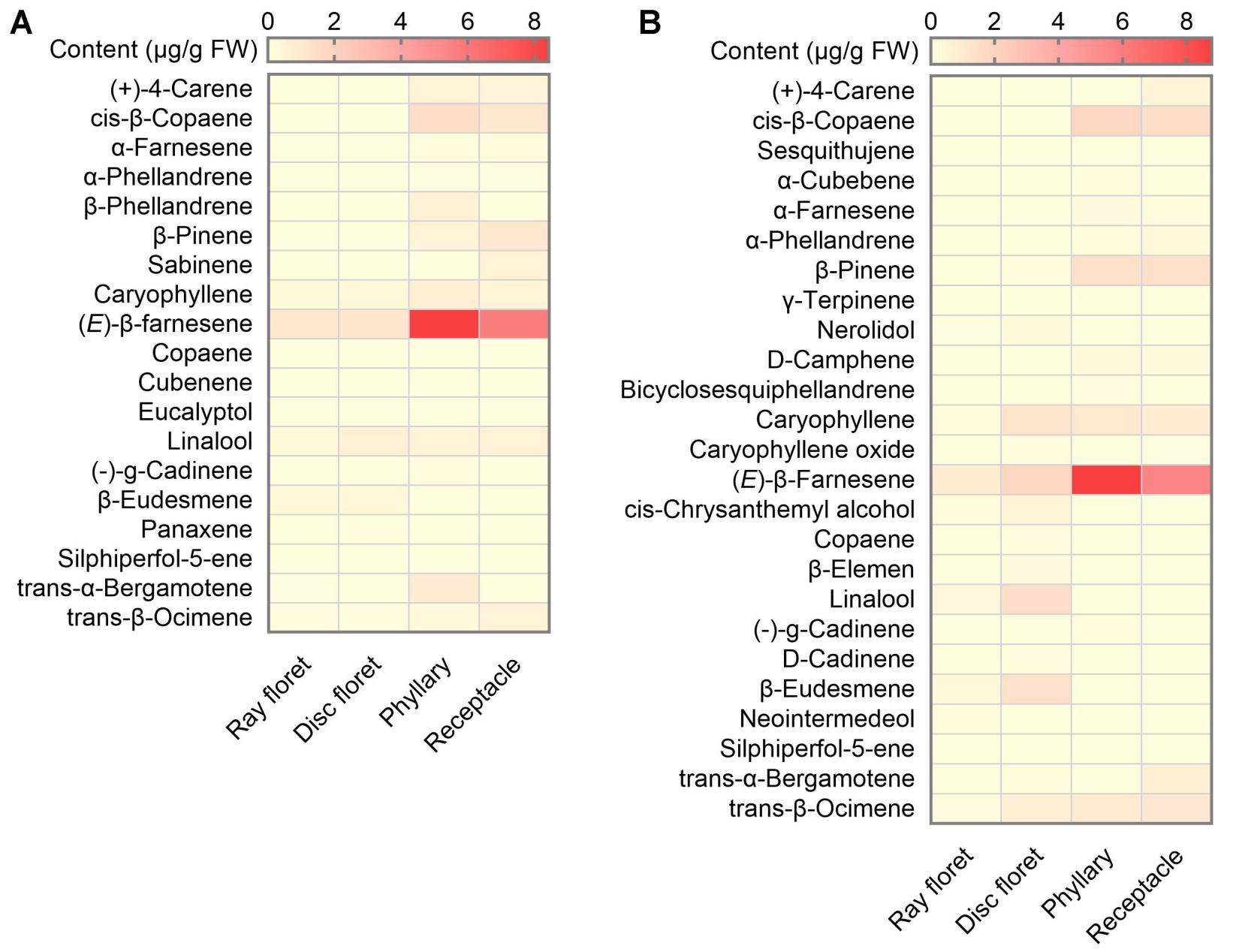


**Supplementary Fig. S3. Analysis of volatile terpenoid content in different floral organs.**

**A)** The heatmap shows the volatile terpenoid content in different floral organs during stage 1 determined by GC-MS. **B)** The heatmap shows the volatile terpenoid content in different floral organs during stage 2 determined by GC-MS. The color scale was used to indicate the content of the VOCs (μg/g FW). For **A) and B)**, the ray floret, disc floret, phyllary, receptacle of 20 flowers were pooled together as one biological replicate. Three biological replicates were performed.


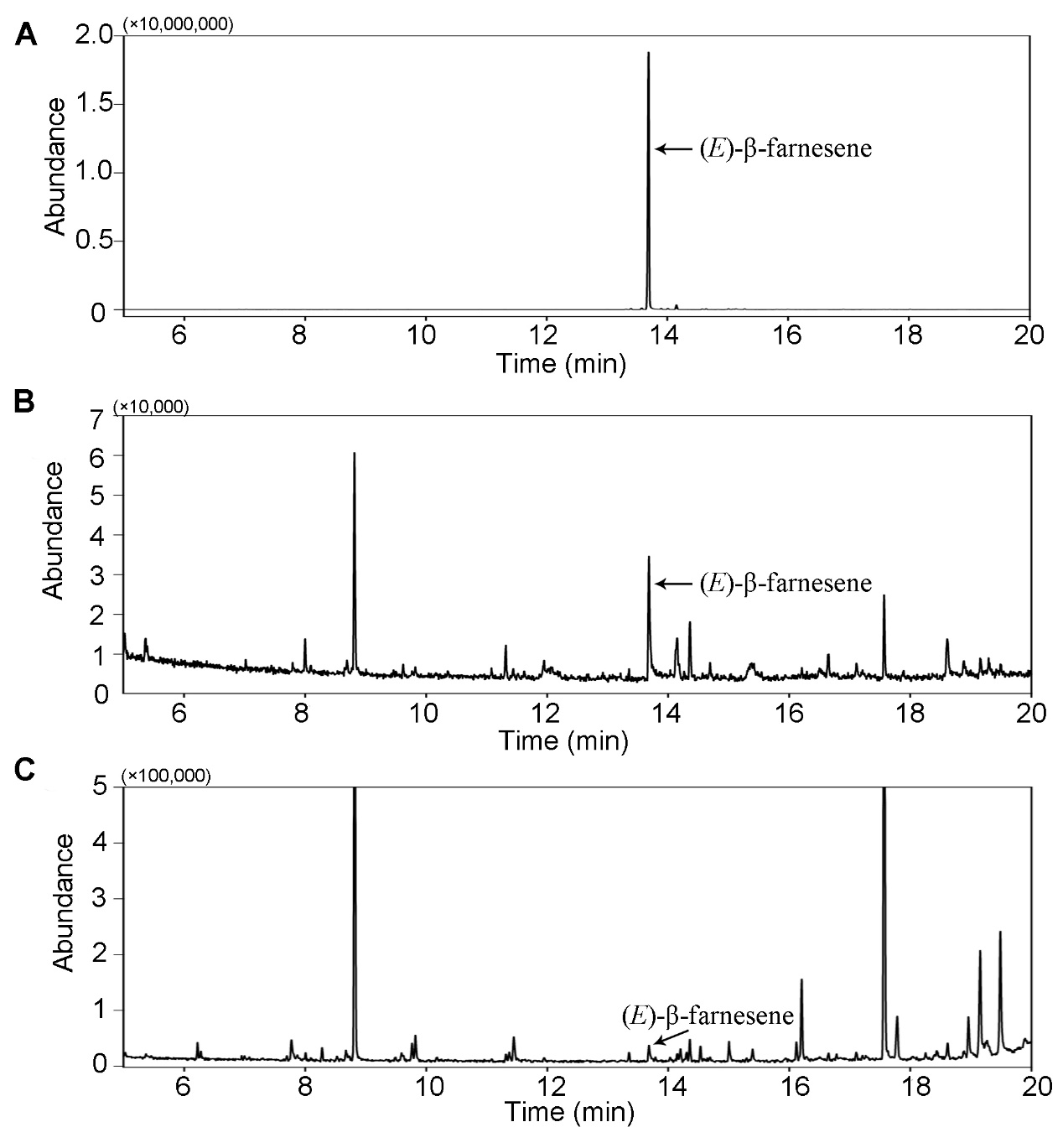


**Supplementary Fig. S4. Detection of VOCs in glands.**

**A)** Product peaks showing the (*E*)-β-farnesene standard. **B)** Detection of VOCs in the glands of the phyllaries. There is a peak at the position of (*E*)-β-farnesene standard, indicating that (*E*)-β-farnesene can be detected in the glands of phyllaries. **C)** Detection of VOCs in the glands of the receptacles. There is a peak at the position of (*E*)-β-farnesene standard, indicating that (*E*)-β-farnesene can be detected in the glands of receptacles.


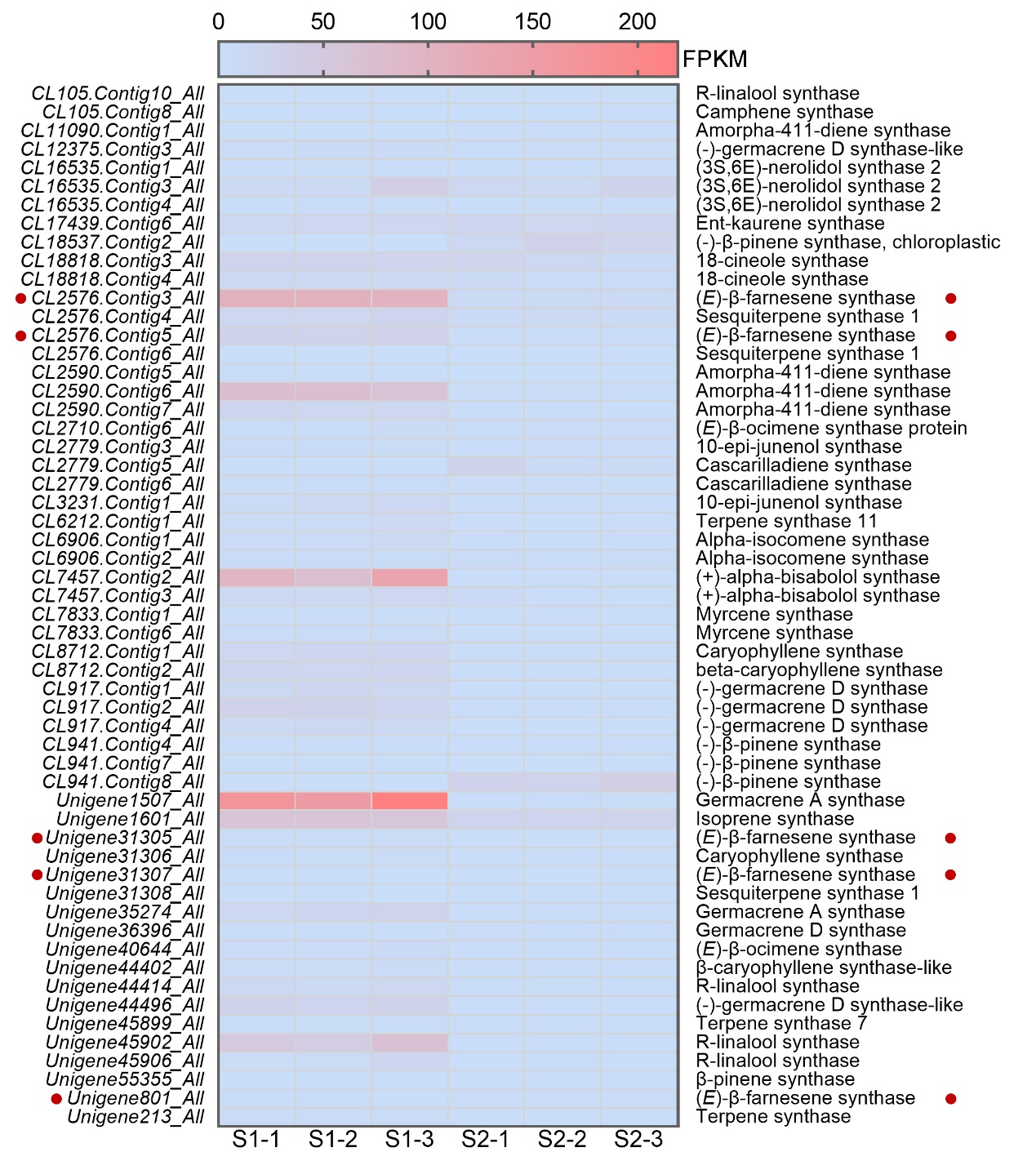


**Supplementary Fig. S5. Transcriptomic analyses of terpene synthase genes.**

The heatmap shows the expression profiles of terpene synthase genes during floral development by RNA-seq. The color scale at the top of the figure represents the FPKM of the gene. The red dots indicate the annotated genes of (*E*)-β-farnesene synthase genes.


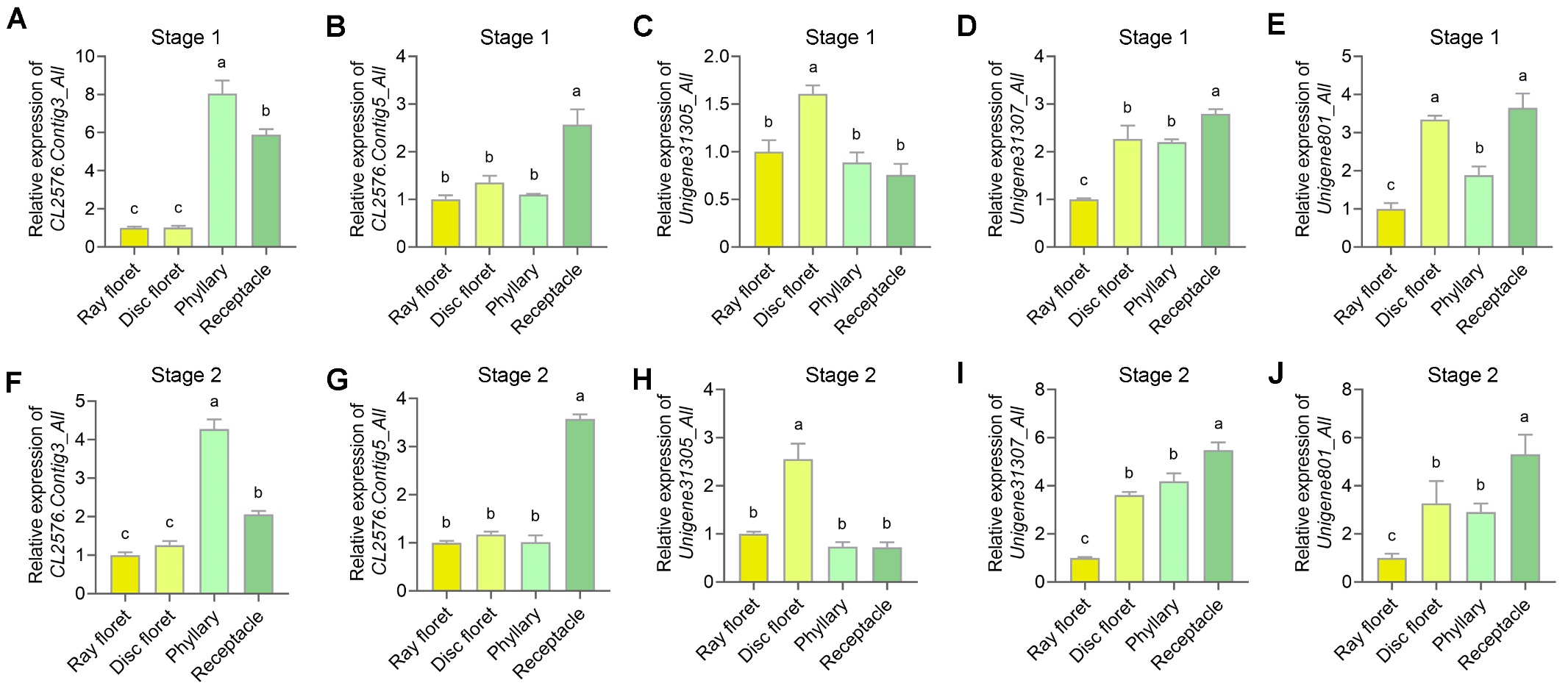


**Supplementary Fig. S6. Tissue-specific expression analysis of (*E*)-β-farnesene synthase genes in chrysanthemum.**

**A)-E)** Expression levels of five candidate genes in different floral organs of chrysanthemum in stage1, as determined by RT-qPCR. **F)-J)** Expression levels of five candidate genes in different floral organs of chrysanthemum during stage 2, as determined by RT-qPCR. *CmUBI* was used as an internal reference. Five flowers were pooled together as one biological replicate. Values are the means ± SD of three biological replicates (n=3). Statistical significance was determined by one-way ANOVA with the Tukey comparisons test (*P* < 0.05).


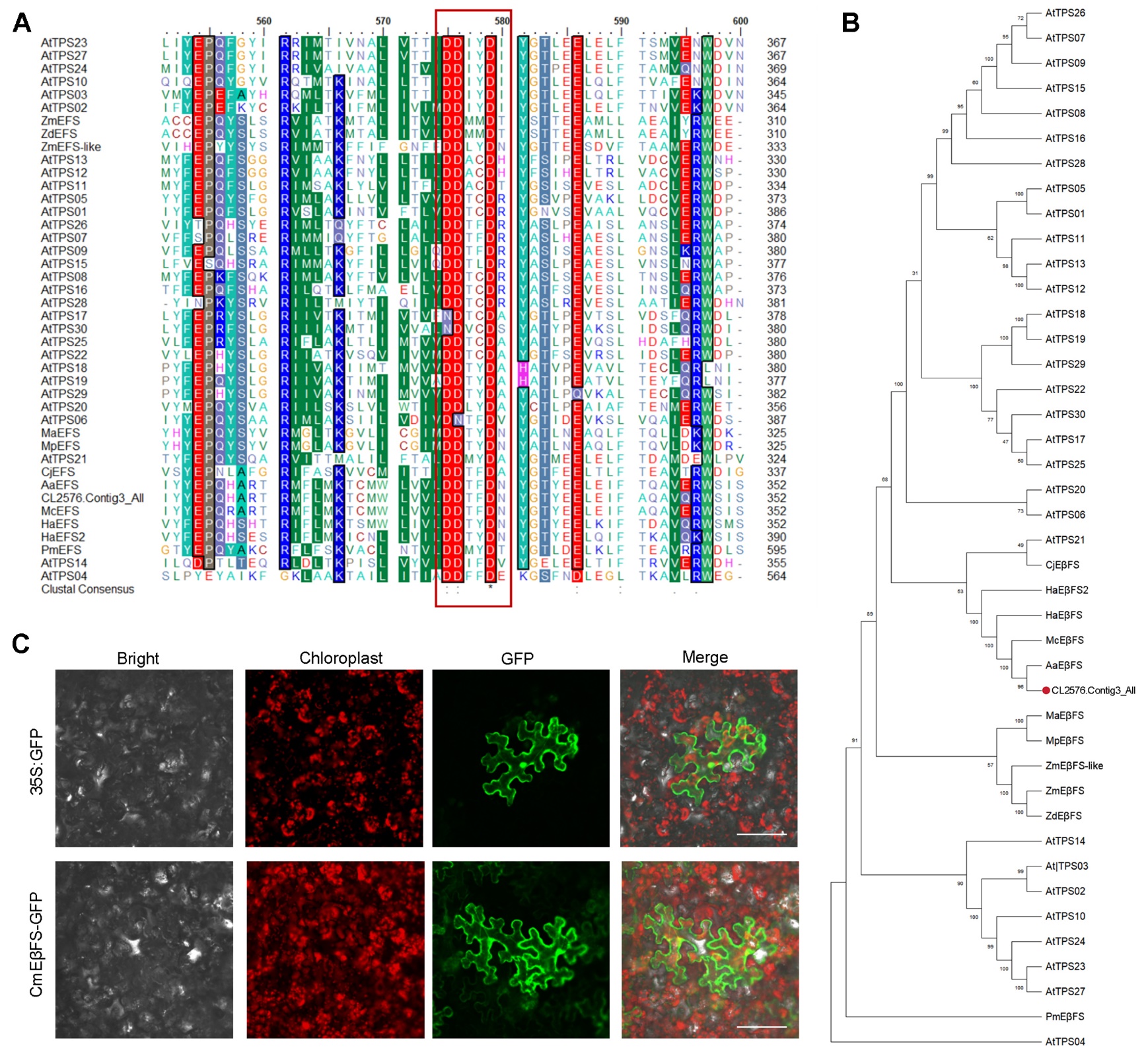


**Supplementary Fig. S7.** **Characterization of CmEβFS.**

**A)** Sequence alignment of the deduced amino acid sequences of TPS homologs. At: *Arabidopsis thaliana*; Pm: *Pseudotsuga menziesii*; Ma: *Mentha arvensis*; Mp: *Mentha*×*piperita*; Zm: *Zea mays*; Zd: *Zea diploperennis*; Cj: *Citrus junos*; Ha: *Helianthus annuus*; Mc: *Matricaria chamomilla*; Aa: *Artemisia annua*. The red box indicates the conserved DDxxD domain of a hallmark of TPS proteins. **B)** Phylogenetic analysis of TPS homologs from various plant species. **C)** Subcellular localization of the CmEβFS-GFP fusion protein in *Nicotiana benthamiana* leaf cells. GFP was fused to the C termini of CmEβFS. Bright, bright-field image; Chloroplast, chlorophyll autofluorescence; GFP, GFP fluorescence; Merged, merged green and chlorophyll autofluorescence. Bar, 100 μm.


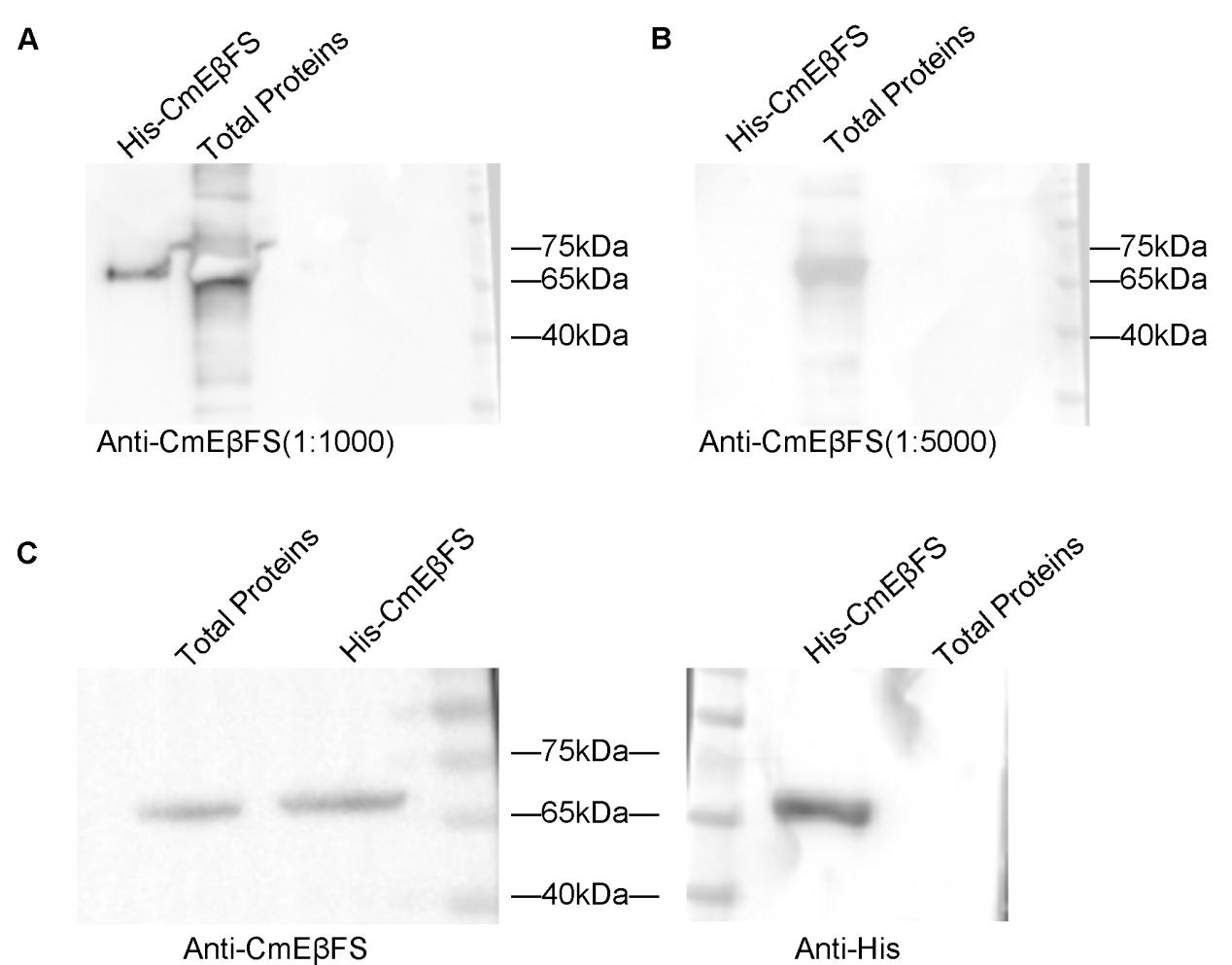


**Supplementary Fig. S8. Analysis of CmEβFS antibody specificity determination.**

**A)** Immunoblot analysis of chrysanthemum total proteins in stage 1 and His-CmEβFS fusion protein using anti-CmEβFS (1:1000). **B)** Immunoblot analysis of chrysanthemum total proteins in stage 1 and His-CmEβFS fusion protein using anti- CmEβFS (1:5000). **C)** Immunoblot analysis of chrysanthemum total proteins in stage 1 and His-CmEβFS fusion protein using anti-CmEβFS (1:1000, left panel) and anti-His (1:1000, right panel).


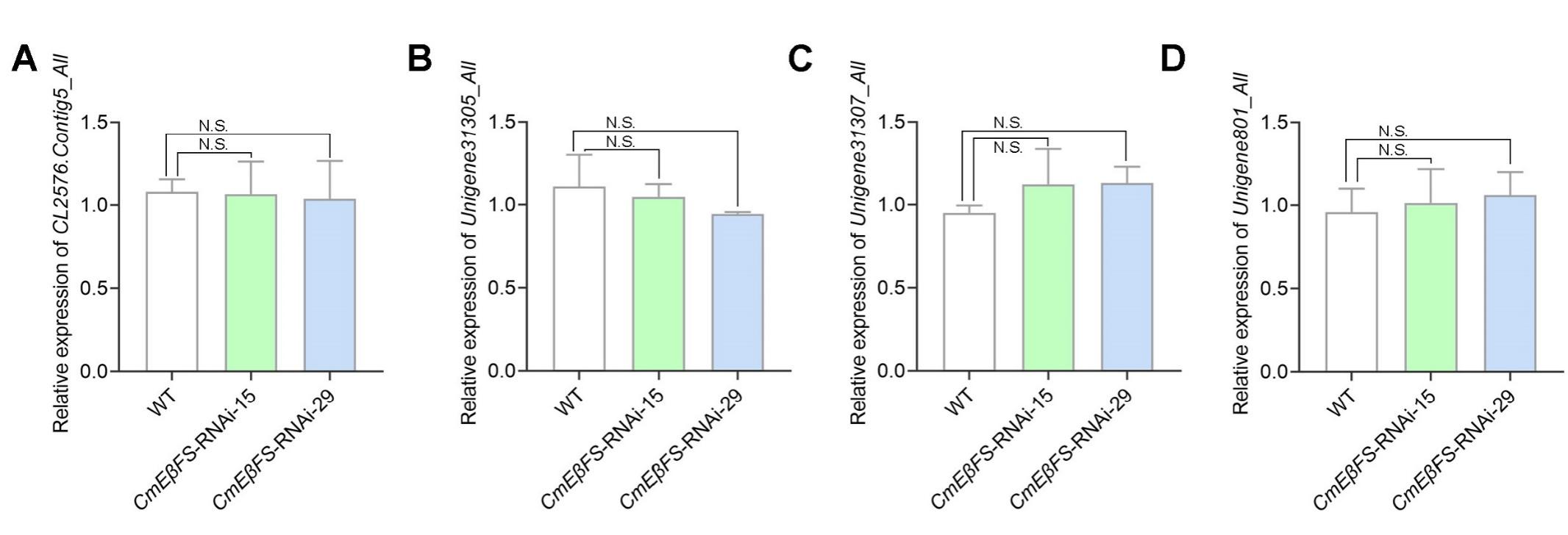


**Supplementary Fig. S9. Expression profiles of candidate (*E*)-β-farnesene synthase genes.**

Relative expression analysis of candidate (*E*)-β-farnesene synthase genes in transgenic plants. **A)-D)** RT-qPCR analysis of *CL2576.Contig 5_All*, *Unigene 31305_All*, *Unigene 31307_All and Unigene 801_All* expression levels in flower of wild type (WT) and *CmEβFS* -RNAi plants. Five flowers at stage 3 were pooled together as one biological replicate. Values are the means ± SD of three biological replicates (n=3). And N.S. means no significance according to Student’s *t* test.

**
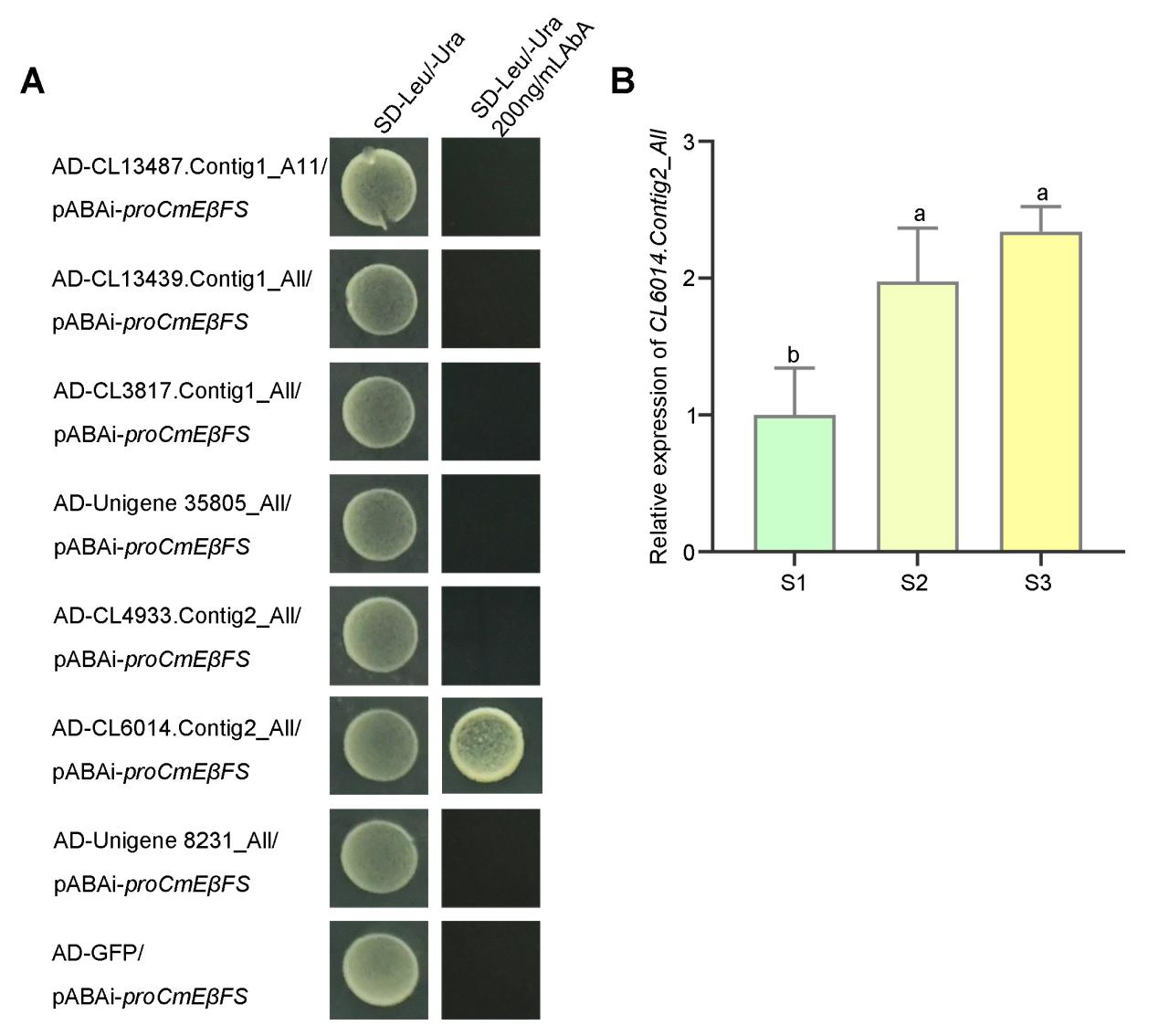
**

**Supplementary Fig. S10. Interaction analysis between candidate transcription factors and the *CmEβFS* promoter.**

**A)** Y1H assay to test for interactions of candidate transcription factors with the *CmEβFS* promoter. **B)** Expression analysis of *CL6014.Contig2_All* during floral development of chrysanthemum. Five flowers were pooled together as one biological replicate at each development stage. Data represent the mean ± SD of three biological replicates (n=3). Statistical significance was assessed using one-way ANOVA analysis of variance (*P* < 0.05).


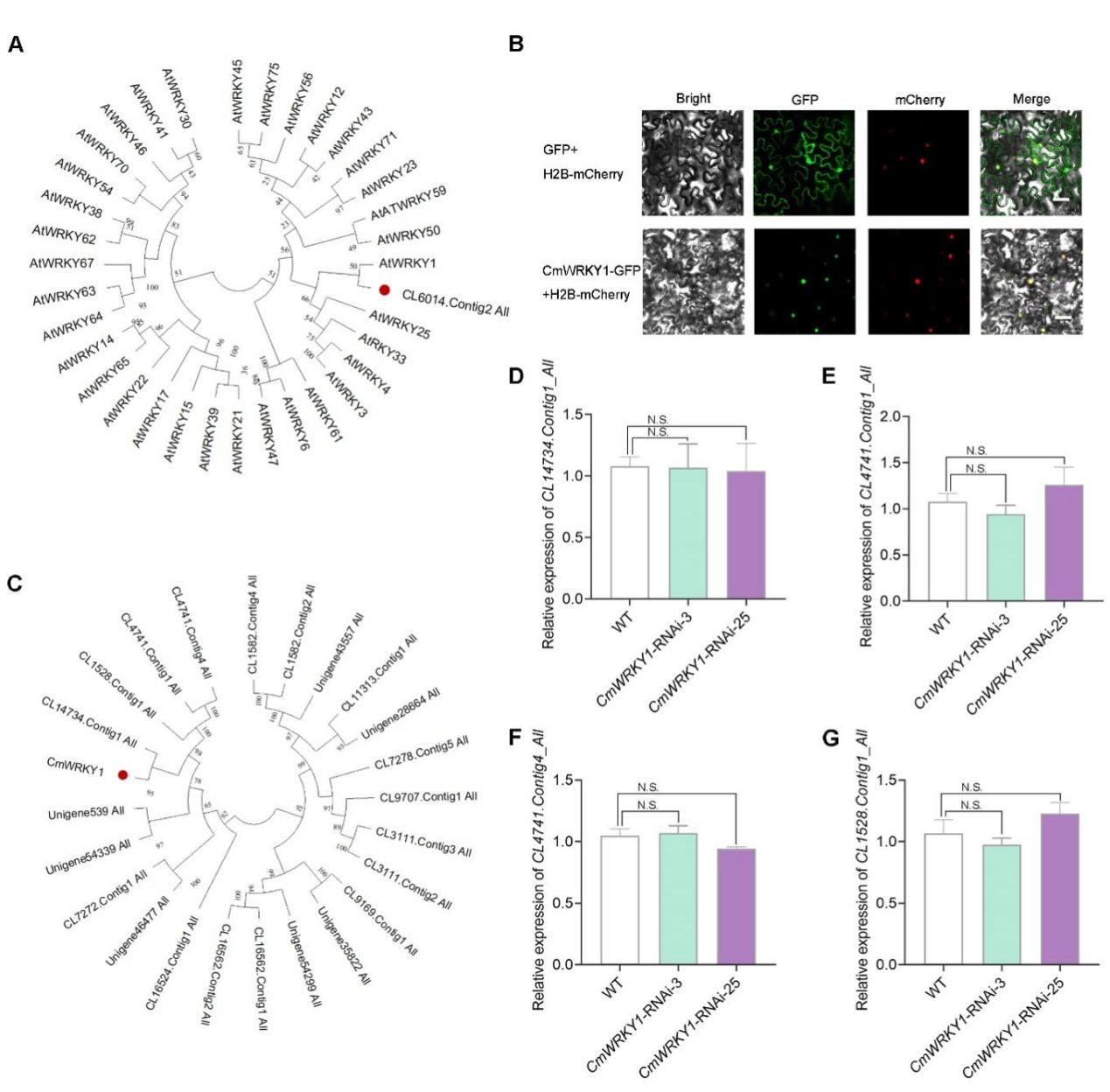


**Supplementary Fig. S11. Characterization of CmWRKY1**

**A)** Phylogenetic analysis of CL6014. Contig2_All (CmWRKY) and WRKY homologs from *Arabidopsis thaliana*. CL6014. Contig2_All (CmWRKY) clustered with AtWRKY1, and named CmWRKY1. **B)** Subcellular localization of CmWRKY1 in *N. benthamiana* leaf cells. CmWRKY1-GFP was co-expressed with the nucleus marker H2B-mCherry. Bright, bright-field image; GFP, GFP fluorescence was observed under 488-nm; mCherry, mCherry fluorescence was observed under 561-nm; Merged, merged green and mCherry. Bars, 50 µm. **C)** Phylogenetic analysis of WRKY identified in the transcriptome of chrysanthemum. CL14734. Contig1_All, CL4741. Contig1_All, CL4741. Contig4_All and CL1528. Contig1_All were cluster with CmWRKY1. **D)-G)** RT-qPCR analysis of *CL14734. Contig1_All*, *CL4741. Contig1_All*, *CL4741. Contig4_All* and *CL1528. Contig1_All* expression levels in flower of wild type (WT) and *CmWRKY1* -RNAi plants. Five flowers at stage 3 were pooled together as one biological replicate. Values are the means ± SD of three biological replicates (n=3). And N.S. means no significance according to Student’s *t* test.


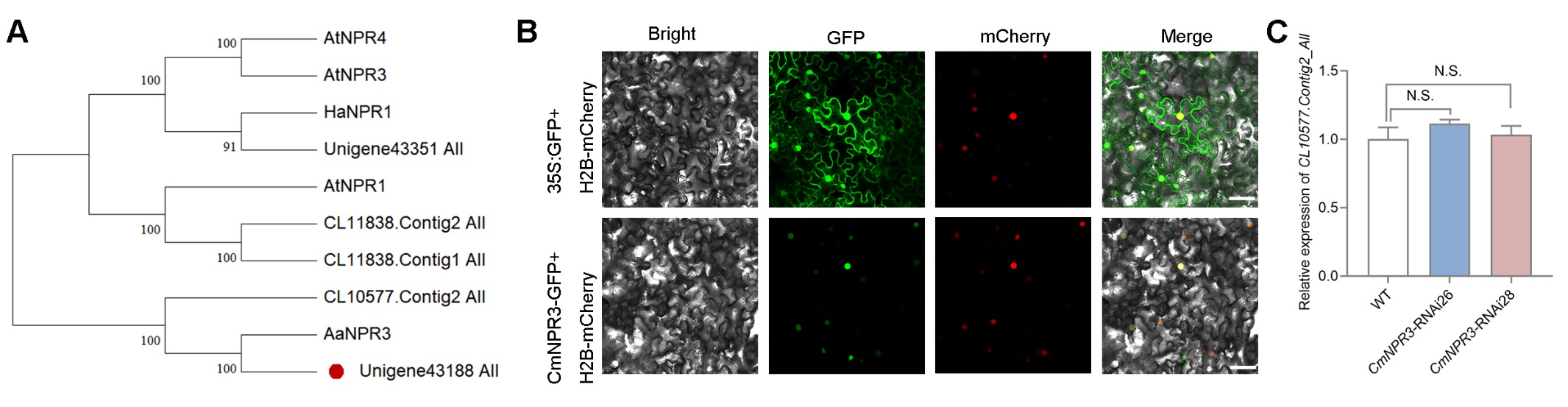


**Supplementary Fig. S12. Characterization of CmNPR3.**

**A)** Phylogenetic analysis of Unigene43188_All (CmNPR) and NPR and from various plant species. Unigene43188_All (CmNPR3) clustered with AaNPR3, named CmNPR3. At: *Arabidopsis thaliana*; Ha: *Helianthus annuus*; Aa: *Artemisia annua*. **B)** Subcellular localization of CmNPR3 in *Nicotiana benthamiana* leaf cells. Bright, bright-field image; GFP, GFP fluorescence was observed under 488-nm; mCherry, mCherry fluorescence was observed under 561-nm; Merged, merged green and mCherry. Bars, 50 µm. **C)** RT-qPCR analysis of *CL10577.Contig2_All* expression levels in flower of wild type (WT) and *CmNPR3*-RNAi. Five flowers at stage 3 were pooled together as one biological replicate. Values are the means ± SD of three biological replicates (n=3). And N.S. means no significance according to Student’s *t* test.


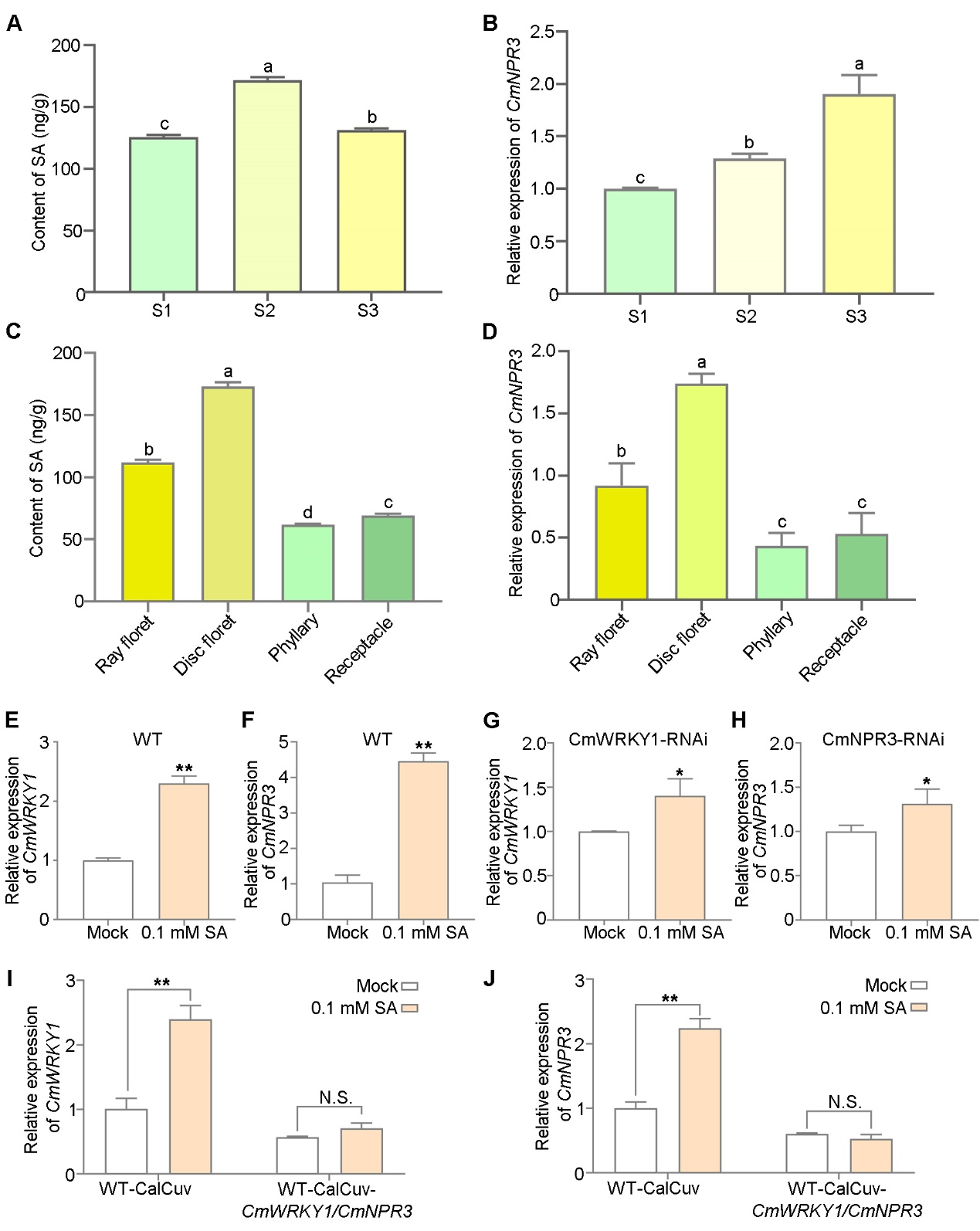


**Supplementary Fig. S13. The effect of SA on the biosynthesis of (*E*)-β-farnesene in chrysanthemum.**

**A)** Changes in SA content during floral development. Each stage was determined for three biological replicates, each consisting of 12 flowers. **B)** RT-qPCR analysis of the expression of SA receptors *CmNPR3* during floral development. Mean values ± SD are shown, n=3. **C)** Changes in SA content in different floral organs. Each organ was determined for three biological replicates, each consisting of 12 flowers. **D)** RT-qPCR analysis of the expression of SA receptors *CmNPR3* in different organs. Mean values ± SD are shown, n=3. Statistical significance was assessed using one-way ANOVA analysis of variance (*P* < 0.05). S1, stage 1; S2, stage 2; S3, stage 3. **E-F)** Expression of *CmWRKY1* and *CmNPR3* following SA treatment of WT flowers at stage 1. **G-H)** Expression of *CmWRKY1* and *CmNPR3* following SA treatment of *CmWRKY1*-RNAi flowers at stage 1. **I-J)** Expression of *CmWRKY1* and *CmNPR3* following SA treatment of WT-CalCuv and WT-CalCuv-*CmWRKY1/CmNPR3* flowers at stage 1. Statistical significance was determined by Student’s *t*-test (**P* < 0.05; ***P* < 0.01).


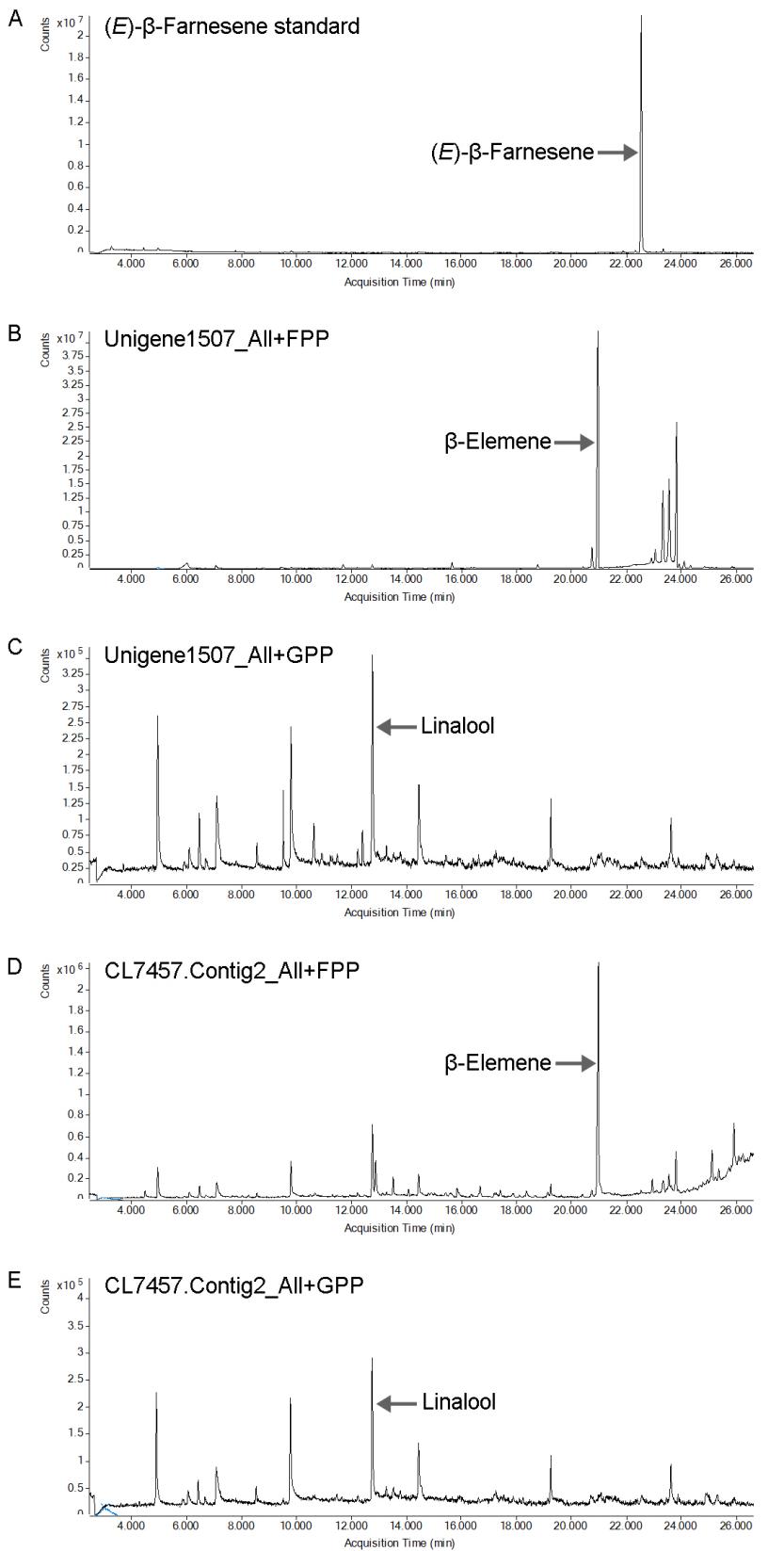


**Supplementary Fig. S14. Identification of enzyme activity products of Unigene1507_All and CL7457.Contig2_All.**

**A)** Product peaks showing the standard of (*E*)-β-farnesene (CATO Research Chemicals Inc., Guangzhou). **B)-C)** The results of enzyme activity assay in vitro for Unigene1507_All using FPP (B) or GPP (C) as substrates. FPP, farnesyl diphosphate. GPP, geranyl diphosphate. Recombinant His- Unigene1507_All proteins purified from *E. coli* were mixed with FPP or GPP. The enzymatic reaction products were analyzed by GC-MS. **D)-E)** The results of enzyme activity assay in vitro for CL7457.Contig2_All using FPP (B) or GPP (C) as substrates. FPP, farnesyl diphosphate. GPP, geranyl diphosphate. Recombinant His- CL7457.Contig2_All proteins purified from *E. coli* were mixed with FPP or GPP. The enzymatic reaction products were analyzed by GC-MS.
