# Supplementary material for: Salicylic acid regulates biosynthesis of floral fragrance (E)-β-farnesene via NPR3-WRKY1 module in chrysanthemum
Source: Mol Hortic. 2025 Sep 5;5:52. doi: 10.1186/s43897-025-00174-y (PMC12412252; doi:10.1186/s43897-025-00174-y)
Supplement: Supplementary file 2 — Supplementary Material 2: Supplementary Table S1. Basic information analysis of transcriptome sequencing. Supplementary Table S2. Information assembled from transcriptome sequencing data. Supplementary Table S3. Seven major functional databases for unigenes annotation. Supplementary Table S4. Candidate transcription factors interacting with CmEβFS promoter. Supplementary Table S5. Analysis of CmWRKY1-interacting proteins. Supplementary Table S6. Proteins used for phylogenetic tree analysis. Supplementary Table S7. CmWRKY1 homologous proteins used for phylogenetic tree analysis. Supplementary Table S8. Primers used in the study. [file 43897_2025_174_MOESM2_ESM.docx]

Supplementary Table S1. Basic information analysis of transcriptome sequencing

| Sample | Total Raw Reads (M) | Total Clean Reads (M) | Total Clean Bases (Gb) | Clean Reads Q20 (%) | Clean Reads Q30 (%) | Clean Reads Ratio (%) |
| --- | --- | --- | --- | --- | --- | --- |
| Stage 1 -1 | 43.82 | 42.89 | 6.43 | 95.69 | 89.94 | \| 97.87 \| \| --- \| |
| Stage 1 -2 | 43.82 | 42.87 | 6.43 | 95.73 | 90.05 | 97.82 |
| Stage 1 -3 | 43.82 | 42.69 | 6.4 | 95.75 | 90.09 | 97.41 |
| Stage 2 -1 | 43.82 | 42.89 | 6.43 | 95.62 | 89.83 | 97.87 |
| Stage 2 -2 | 45.57 | 42.47 | 6.37 | 95.88 | 90.45 | 93.19 |
| Stage 2 -3 | 43.82 | 42.81 | 6.43 | 95.66 | 89.92 | 97.7 |

Supplementary Table S2. Information assembled from transcriptome sequencing data

| Sample | Total Number | Total Length | Mean Length | N50 | N70 | N90 | GC (%) |
| --- | --- | --- | --- | --- | --- | --- | --- |
| Stage 1 -1 | 94329 | 84191640 | 892 | 1324 | 854 | 401 | 39.68 |
| Stage 1 -2 | 94057 | 83090206 | 883 | 1303 | 843 | 399 | 39.64 |
| Stage 1 -3 | 95619 | 84935503 | 888 | 1314 | 847 | 398 | 39.66 |
| Stage 2 -1 | 96950 | 84205046 | 868 | 1284 | 825 | 390 | 39.48 |
| Stage 2 -2 | 95987 | 84116042 | 876 | 1296 | 834 | 391 | 39.61 |
| Stage 2 -3 | 92944 | 83594630 | 899 | 1320 | 860 | 407 | 39.65 |

Supplementary Table S3. Seven major functional databases for unigenes annotation

| stat_values | stat_total | stat_nr | stat_nt | stat_swissprot | stat_kegg | stat_kog | stat_pfam | stat_go |
| --- | --- | --- | --- | --- | --- | --- | --- | --- |
| Number | 166,324 | 109,252 | 67,935 | 73,846 | 78,957 | 76,976 | 66,771 | 85,048 |
| Percentage | 100% | 65.69% | 40.84% | 44.40% | 47.47% | 46.28% | 40.15% | 51.13% |

Supplementary Table S4. Candidate transcription factors interacting with *CmEβFS* promoter

| Gene | Annotation |
| --- | --- |
| CL3817.Contig1_All | MADS-box transcription factor CDM44 |
| Unigene35805_All | bHLH-MYC and R2R3-MYB transcription factors N-terminal |
| CL13487.Contig1_All | MADS-box transcription factor CDM104 |
| Unigene8231_All | hypothetical protein CTI12 |
| CL4933.Contig2_All | transcription factor TCP2-like |
| CL6014.Contig2_All | WRKY transcription factor |
| CL13439.Contig1_All | NAC domain containing protein 52-like |

Supplementary Table S5. Analysis of CmWRKY1-interacting proteins

| ID | Annotation |
| --- | --- |
| CL12750.Contig1_All | GTP binding Elongation factor Tu family protein [Artemisia annua] |
| CL1245.Contig1_All | ribulose-1,5-bisphosphate carboxylase/oxygenase large subunit (chloroplast) [Chrysanthemum × morifolium] >AFA45282.1 ribulose-1,5-bisphosphate carboxylase/oxygenase large subunit (chloroplast) [Chrysanthemum × morifolium] >APU51374.1 ribulose 1,5-bisphosphate carboxylase/oxygenase large subunit (plastid) [Chrysanthemum x morifolium] |
| CL51.Contig4_All | putative histone H2B.3 [Artemisia annua] |
| CL2046.Contig4_All | lipid transfer protein-4 [Artemisia annua] >PWA45198.1 non-specific lipid-transfer protein [Artemisia annua] >PWA59758.1 non-specific lipid-transfer protein [Artemisia annua] |
| Unigene43188_All | Ankyrin repeat-containing protein [Artemisia annua] >QAV82484.1 NPR3 [Artemisia annua] |
| CL18535.Contig17_All | SKP1/BTB/POZ domain, NPH3 domain protein [Artemisia annua] |
| CL17006.Contig9_All | uncharacterized protein LOC110803438 [Spinacia oleracea] |
| CL8658.Contig2_All | glyceraldehyde-3-phosphate dehydrogenase of plastid 2 [Artemisia annua] |
| Unigene49440_All | 12-oxophytodienoate reductase 1-like [Cynara cardunculus var. scolymus] |
| Unigene28186_All | heat shock factor protein HSF8 [Helianthus annuus] >OTG22519.1 putative heat shock factor protein HSF8 [Helianthus annuus] |
| CL4218.Contig1_All | hypothetical protein CTI12_AA339080 [Artemisia annua] |
| CL5279.Contig2_All | Cobalamin-independent methionine synthase [Artemisia annua] |
| CL16204.Contig1_All | acetyl-coenzyme A synthetase, chloroplastic/glyoxysomal-like [Helianthus annuus] >XP_021973449.1 acetyl-coenzyme A synthetase, chloroplastic/glyoxysomal-like [Helianthus annuus] >XP_021973450.1 acetyl-coenzyme A synthetase, chloroplastic/glyoxysomal-like [Helianthus annuus] |
| CL14999.Contig2_All | chaperone protein ClpB4, mitochondrial-like [Lactuca sativa] >PLY81716.1 hypothetical protein LSAT_3X21960 [Lactuca sativa] |
| CL4022.Contig1_All | chaperone protein ClpB [Artemisia annua] |
| Unigene43413_All | cold shock protein 2-like [Helianthus annuus] >OTF86721.1 putative zinc finger, CCHC-type, Cold shock, CspA [Helianthus annuus] |
| CL18989.Contig2_All | recA DNA recombination family protein [Artemisia annua] |
| CL6710.Contig1_All | carbamoyl-phosphate synthase B [Artemisia annua] |
| Unigene28961_All | GDSL esterase/lipase At4g28780-like [Cynara cardunculus var. scolymus] >KVI12315.1 Lipase, GDSL [Cynara cardunculus var. scolymus] |
| CL3325.Contig3_All | aquaporin PIP2 [Chrysanthemum × morifolium] |
| Unigene1391_All | ribosomal protein L6 family [Artemisia annua] |
| CL7060.Contig5_All | ribulose-1,5-bisphosphate carboxylase small subunit [Chrysanthemum × morifolium] |
| CL2975.Contig1_All | beta-tubulin [Chrysanthemum indicum] |
| CL16896.Contig2_All | Polyphenol oxidase [Artemisia annua] |
| Unigene1573_All | glyceraldehyde-3-phosphate dehydrogenase-like family protein [Artemisia annua] |
| CL6602.Contig2_All | Zinc finger, RING/FYVE/PHD-type [Artemisia annua] |
| CL7558.Contig1_All | ribonuclease D, Exosome-associated factor Rrp6 [Artemisia annua] |
| Unigene43168_All | luminal-binding protein 5-like [Helianthus annuus] |
| CL6101.Contig8_All | hypothetical protein CTI12_AA082610 [Artemisia annua] |
| CL15874.Contig1_All | small and basic intrinsic protein 2,1 [Artemisia annua] |
| Unigene25135_All | puromycin-sensitive aminopeptidase-like isoform X1 [Lactuca sativa] |
| CL18403.Contig1_All | light-harvesting chlorophyll a/b-binding protein (LHCP) precursor [Artemisia annua] |
| CL6891.Contig1_All | TRAF-like, SKP1/BTB/POZ domain, BTB/Kelch-associated [Artemisia annua] |
| Unigene28606_All | Ankyrin repeat-containing protein [Artemisia annua] |
| CL13828.Contig1_All | dihydrolipoamide succinyltransferase [Artemisia annua] |
| CL6613.Contig1_All | aluminum induced protein [Artemisia annua] |
| CL3386.Contig2_All | probable U6 snRNA-associated Sm-like protein LSm4 [Solanum lycopersicum] |
| CL16601.Contig1_All | adenylate kinase/UMP-CMP kinase, P-loop containing nucleoside triphosphate hydrolase [Artemisia annua] |

Supplementary Table S6. Proteins used for phylogenetic tree analysis

| Protein | ID |
| --- | --- |
| AtTPS01 | O23651.1 |
| AtTPS02 | P0CJ43.1 |
| AtTPS03 | A4FVP2.1 |
| AtTPS04 | Q93YV0.1 |
| AtTPS05 | O22184.2 |
| AtTPS06 | Q84UU9.2 |
| AtTPS07 | O65434.2 |
| AtTPS08 | O65435.2 |
| AtTPS09 | Q8L7G4.2 |
| AtTPS10 | Q9ZUH4.1 |
| AtTPS11 | Q4KSH9.2 |
| AtTPS12 | Q9T0J9.2 |
| AtTPS13 | Q9T0K1.2 |
| AtTPS14 | Q84UV0.2 |
| AtTPS15 | Q9LS76.2 |
| AtTPS16 | Q9LVP7.1 |
| AtTPS17 | Q9LRR2.2 |
| AtTPS18 | Q9LUE2.1 |
| AtTPS19 | Q9LUE0.1 |
| AtTPS20 | Q9FI27.3 |
| AtTPS21 | Q84UU4.2 |
| AtTPS22 | Q9LQ27.2 |
| AtTPS23 | P0DI77.1 |
| AtTPS24 | Q9LRZ6.1 |
| AtTPS25 | Q9LIA1.2 |
| AtTPS26 | Q9C8E3.1 |
| AtTPS27 | P0DI76.1 |
| AtTPS28 | Q9C748.1 |
| AtTPS29 | Q9C6W6.2 |
| AtTPS30 | Q9LH31.1 |
| AaEβFS | ADT64306.1 |
| CjEβFS | Q94JS8.1 |
| HaEβFS | KAF5761491.1 |
| HaEβFS2 | KAF5753671.1 |
| MaEβFS | ADC92564.1 |
| McEβFS | AIW60869.1 |
| MpEβFS | AEK32002.1 |
| PmEβFS | ADX42739.1 |
| ZdEβFS | C7E5V9.1 |
| ZmEβFS | C7E5V8.1 |
| ZmEβFS-like | NP_001351578.1 |
| AtWRKY1 | AT2G04880.1 |
| AtWRKY3 | AT2G03340.1 |
| AtWRKY4 | AT1G13960.1 |
| AtWRKY6 | AT1G62300.1 |
| AtWRKY12 | AT2G44745.1 |
| AtWRKY14 | AT1G30650.1 |
| AtWRKY15 | AT2G23320.1 |
| AtWRKY17 | AT2G24570.1 |
| AtWRKY21 | AT2G30590.1 |
| AtWRKY22 | AT4G01250.1 |
| AtWRKY23 | AT2G47260.1 |
| AtWRKY25 | AT2G30250.1 |
| AtWRKY30 | AT5G24110.1 |
| AtWRKY33 | AT2G38470.1 |
| AtWRKY38 | AT5G22570.1 |
| AtWRKY39 | AT3G04670.1 |
| AtWRKY41 | AT4G11070.1 |
| AtWRKY43 | AT2G46130.1 |
| AtWRKY45 | AT3G01970.1 |
| AtWRKY46 | AT2G46400.1 |
| AtWRKY47 | AT4G01720.1 |
| AtWRKY50 | AT5G26170.1 |
| AtWRKY54 | AT2G40750.1 |
| AtWRKY56 | AT1G64000.1 |
| AtWRKY59 | AT2G21900.1 |
| AtWRKY61 | AT1G18860.1 |
| AtWRKY62 | AT5G01900.1 |
| AtWRKY63 | AT1G66600.1 |
| AtWRKY64 | AT1G66560.1 |
| AtWRKY65 | AT1G29280.1 |
| AtWRKY67 | AT1G66550.1 |
| AtWRKY70 | AT3G56400.1 |
| AtWRKY71 | AT1G29860.1 |
| AtWRKY75 | AT5G13080.1 |
| AtNPR1 | NP_176610.1 |
| AtNPR3 | OAO93003.1 |
| AtNPR4 | NP_193701.2 |
| AaNPR3 | QAV82484.1 |
| HaNPR1 | XP_022001347.2 |

Supplementary Table S7. CmWRKY1 homologous proteins used for phylogenetic tree analysis

| ID | Annotation |
| --- | --- |
| Unigene54339_All | WRKY transcription factor 33 |
| Unigene54299_All | A WRKY superfamily transcription factors |
| Unigene539_All | WRKY transcription factor 2 factor 2 |
| Unigene46477_All | Probable WRKY transcription factor 26 |
| Unigene43557_All | Probable WRKY transcription factor 39 |
| Unigene35822_All | Probable WRKY transcription factor 46 |
| Unigene28664_All | Probable WRKY transcription factor 7 |
| CL9707.Contig1_All | WRKY transcription factor WRKY51 |
| CL9169.Contig1_All | WRKY transcription factor 70 |
| CL7278.Contig5_All | WRKY transcription factor WRKY51 |
| CL7272.Contig1_All | WRKY transcription factor WRKY24 |
| CL4741.Contig4_All | WRKY transcription factor |
| CL4741.Contig1_All | Probable WRKY transcription factor 34 |
| CL3111.Contig2_All | Probable WRKY transcription factor 17 |
| CL16562.Contig2_All | WRKY DNA-binding transcription factor 70 |
| CL16562.Contig1_All | Probable WRKY transcription factor 70 |
| CL16524.Contig1_All | WRKY transcription factor 22 |
| CL1582.Contig4_All | Probable WRKY transcription factor 74 |
| CL1582.Contig2_All | Probable WRKY transcription factor 21 |
| CL1528.Contig1_All | Probable WRKY transcription factor 20 |
| CL14734.Contig1_All | Probable WRKY transcription factor 33 |
| CL11313.Contig1_All | Probable WRKY transcription factor 15 |
| CL3111.Contig3_All | WRKY transcription factor WRKY51 |

Supplementary Table S8. Primers used in the study

| Primer name | Primer |
| --- | --- |
| qRT-*CmEβFS*-F | TTGCTCTGTATGAAGCGGCG |
| qRT-*CmEβFS*-R | CAGCCTTCTCCTAAGTGGCT |
| *CmEβFS*-E1-F | gtatggctagcgaattggcccttATGTCAACTACTATTCCTG |
| *CmEβFS*-E1-R | cgttgagctcgaattggcccttTTAGATGACCATAGGGTGAATG |
| *CmEβFS*-promoter1-F | cttgaattcgagctcggtaccCTGGTGAATGATGTCGTGTGCA |
| *CmEβFS*-promoter1-R | atacagagcacatgcctcgagACTTACCTTCATACTAGCACATTCATCTC |
| *CmEβFS*-promoter2-F | cttgaattcgagctcggtaccACCCTGAAGTTTAGTAAGTATGATTGTTC |
| *CmEβFS*-promoter2-R | atacagagcacatgcctcgagCAAATACTTCAGAATAACCAAACACTTAA |
| *CmEβFS-*promoter3-F | cttgaattcgagctcggtaccCGTTTGTGTCATTAATGTGCTCTG |
| *CmEβFS*-promoter3-R | atacagagcacatgcctcgagCAACCCGACAAGGGTAAAGATATC |
| *CmEβFS*-promoter4-F | cttgaattcgagctcggtaccGATTTATCTCAAATATCTAAGTTTTTAGTGGT |
| *CmEβFS*-promoter4-R | atacagagcacatgcctcgagCTCAAAATCTTGCAAGTTTGATTGA |
| *CmEβFS*-promoter-F | CTGGTGAATGATGTCGTGTGC |
| *CmEβFS*-promoter-R | CTCAAAATCTTGCAAGTTTG |
| *CmEβFS*-LUC-F | gtcgacggtatcgataagcttCTGGTGAATGATGTCGTGTGC |
| *CmEβFS*-LUC-R | cgctctagaactagtggatccCTCAAAATCTTGCAAGTTTG |
| *CmEβFS*-RNAi-F1 | cgatctctttgatggggatccTGCCAATATAATCACATAGTTTGTGG |
| *CmEβFS*-RNAi-R1 | gactctagggactagttaattaaTTTGTGGGCTTGTTGCAGC |
| *CmEβFS*-RNAi-F2 | ttacaattaccatggggcgcgccTTTGTGGGCTTGTTGCAGC |
| *CmEβFS*-RNAi-R2 | catgttcatctggggatttaaatTGCCAATATAATCACATAGTTTGTGG |
| *CmEβFS*-GFP-F | ccaaatcgactctagtctagaATGTCAACTACTATTCCTGTTTCTAGTGTT |
| *CmEβFS*-GFP-R | gcccttgctcaccatggtaccGATGACCATAGGGTGAATGAAGA |
| qRT-*CmWRKY1*-F | AGACCTACTGAGTCTGGTGC |
| qRT-*CmWRKY1*-R | AGGGTAGAACAATAGGCGAGGT |
| *CmWRKY1*-GFP-F | ccaaatcgactctagtctagaATGATGGATAAAACACCGGAA |
| *CmWRKY1*-GFP-R | gcccttgctcaccatggtaccGGTAGCACTAACGTGAAGAAC |
| *CmWRKY1*-SK-F | tcccccgggctgcaggaattcATGATGGATAAAACACCGGAAAA |
| *CmWRKY1*-SK-R | tgatttcagcgaattggtaccTCAGGTAGCACTAACGTGAAGAACC |
| *CmWRKY1*-BD-F | atggccatggaggccgaattcATGATGGATAAAACACCGGAAAA |
| *CmWRKY1*-BD-R | atgcggccgctgcaggtcgacTCAGGTAGCACTAACGTGAAGAACC |
| *CmWRKY1*-YNE-F | gagaacacgggggactctagaATGATGGATAAAACACCGGAAAA |
| *CmWRKY1*-YNE-R | ctccatcccgggagcggtaccGGTAGCACTAACGTGAAGAACCATA |
| *CmWRKY1*-E1-F | gtatggctagcgaattggcccttATGATGGATAAAACACCGG |
| *CmWRKY1*-E1-R | cgttgagctcgaattggcccttGGTTTCTCAGGTAGTTTTG |
| *CmWRKY1*-Flag-F | ccaaatcgactctagtctagaATGATGGATAAAACACCGGAA |
| *CmWRKY1*-Flag-R | gtctttgtagtccatggtaccGGTAGCACTAACGTGAAGAAC |
| *CmWRKY1*-RNAi-F1 | cgatctctttgatggggatccGGGCGATTGATTTAGCGGCCGCG |
| *CmWRKY1*-RNAi-R1 | gactctagggactagttaattaaGGCTTTCGATGGCGCAAGTATG |
| *CmWRKY1*-RNAi-F2 | acatttacaattaccatggggcgcgccGGCTTTCGATGGCGCAAG |
| *CmWRKY1*-RNAi-R2 | atgccatgttcatctggggatttaaatGGGCGATTGATTTAGCGGC |
| *CmNPR3*-RNAi-F1 | CGATCTCTTTGATGGGGATCCATACTCATGATGAGAATGGTGG |
| *CmNPR3*-RNAi-R1 | GACTCTAGGGACTAGTTAATTAACCTCAGATTTCCTATTCAA |
| *CmNPR3*-RNAi-F2 | ACAATTACCATGGGGCGCGCCCCTCAGATTTCCTATTCAAAGG |
| *CmNPR3*-RNAi-R2 | CATGTTCATCTGGGGATTTAAATATACTCATGATGAGAATGG |
| *CmNPR3*-GFP--F | caccaaatcgactctagtctagaATGTCTCTTGAAGATTCATTG |
| *CmNPR3*-GFP-R | tcgcccttgctcaccatggtaccATACTCATGATGAGAATGGT |
| *CmNPR3*-AD-F | gccatggaggccagtgaattcATGTCTCTTGAAGATTCATTG |
| *CmNPR3*-AD-R | cagctcgagctcgatggatccCTAATACTCATGATGAGAATGG |
| *CmNPR3*-SK-F | tcccccgggctgcaggaattcATGTCTCTTGAAGATTCATTG |
| *CmNPR3*-SK-R | tgatttcagcgaattggtaccCTAATACTCATGATGAGAATGG |
| *CmNPR3*-YCE-F | gagaacacgggggactctagaATGTCTCTTGAAGATTCATTGAGATCTT |
| *CmNPR3*-YCE-R | gtacatcccgggagcggtaccATACTCATGATGAGAATGGTGGTAC |
| *CmNPR3*-pGEX-4T-F | gatctggttccgcgtggatccATGTCTCTTGAAGATTCATTG |
| *CmNPR3*-pGEX-4T-R | ctcgagtcgacccgggaattcCTAATACTCATGATGAGAATG |
| *CmNPR3*-Myc-F | gatacaccaaatcgactctagaATGTCTCTTGAAGATTCATT |
| *CmNPR3*-Myc-R | gagcttttgctccatggtaccATACTCATGATGAGAATGGTG |
| *CL10577.Contig2*-qRT-F | GGCATACACATTGCACCTCTG |
| *CL10577.Contig2*-qRT-R | GCTTCTGAGTGAGTAGCACAAG |
| *CmWRKY1*-ChIP-P1-F | TGATGTCGTGTGCAATACGC |
| *CmWRKY1*-ChIP-P1-R | AACTACCACGAGCCCAGAAC |
| *CmWRKY1*-ChIP-P2-F | TGATTCACAAGCTGTCATCCGA |
| *CmWRKY1*-ChIP-P2-R | CGTATGGGCAAAGTTCACCAGT |
| *CmWRKY1*-ChIP-P3-F | TCCGCTGCTACAATGCTGAA |
| *CmWRKY1*-ChIP-P3-R | AGCACATTAATGACACAAACGCA |
| *CmWRKY1*-ChIP-P4-F | TGCTTCCGCAGAATCCTCAA |
| *CmWRKY1*-ChIP-P4-R | TGCTTAATACCTGCCGCAGAA |
| *CmWRKY1*-ChIP-P5-F | CAAGATGAGCTTATGGGATAGC |
| *CmWRKY1*-ChIP-P5-R | CTATACAAAAGCGGAAGCCT |
| *CmWRKY1*-ChIP-P6-F | GTTGGCAATGGCTATATAAACACGA |
| *CmWRKY1*-ChIP-P6-R | AGTACAGGTTCATGTGCATCTCA |
| *CL14734.Contig1*-qRT-F | GTGTACTCATGCTAACTGTC |
| *CL14734.Contig1*-qRT-R | GTCGCTCGACGATCCAAGCCC |
| *CL4741.Contig1*-qRT-F | GGGAATGTTCAAGACCATCA |
| *CL4741.Contig1*-qRT-R | CTTCTTGGATACTCACTACCC |
| *CL4741.Contig4*-qRT-F | GGAACCCTCGATATTACTCC |
| *CL4741.Contig4*-qRT-R | GCCACGCACAACTTTCTGGC |
| *CL1528.Contig1*-qRT-F | GGGTGGAAGGTGAAACAATA |
| *CL1528.Contig1*-qRT-R | GTGGCTGCTTTAACGCTTGG |
| *CL2576.Contig5*-qRT-F | GTGTACTCATGCTAACTGTC |
| *CL2576.Contig5*-qRT-R | GTCGCTCGACGATCCAAGCCC |
| *Unigene31305*-qRT-F | CCAACGCCTTGGCATAGCCT |
| *Unigene31305*-qRT-R | CTTGTTGCCTTAGGACTCGG |
| *Unigene31307*-qRT-F | GGCTCTAATGAACCAATGCA |
| *Unigene31307*-qRT-R | TTATCGACCCACTGCTCGCC |
| *Unigene801*-qRT-F | GAGAGATGATGTTCGAGGCA |
| *Unigene801*-qRT-R | GAGCTTCAAGAGAACTGTCG |
| *Unigene1507*-F | CTAGTCCGATCCACCTTCAAG |
| *Unigene1507*-R | TGCAACGGTTAAGCGTTGTCG |
| *Unigene1507*-pET28-F | atgggtcgcggatccgaattcATGGCAACGGTTCAAGCTAATG |
| *Unigene1507*-pET28-R | tgcggccgcaagcttgtcgacTTACGCGGGTAGAGAACCCAC |
| *CL7457.Contig2*-F | GGTGCGGCAGCTACTTAAAG |
| *CL7457.Contig2*-R | CAACAGTGGCCTTGGAATGG |
| *CL7457.Contig2*-pET28-F | atgggtcgcggatccgaattcATGAAACATGCCAATTTGCTAAAG |
| *CL7457.Contig2*-pET28-R | tgcggccgcaagcttgtcgacTCATATACTCATAGGATAAACGAGTAG |
